# Integrated gut/liver microphysiological systems elucidates inflammatory inter‐tissue crosstalk

**DOI:** 10.1002/bit.26370

**Published:** 2017-07-27

**Authors:** Wen L.K. Chen, Collin Edington, Emily Suter, Jiajie Yu, Jeremy J. Velazquez, Jason G. Velazquez, Michael Shockley, Emma M. Large, Raman Venkataramanan, David J. Hughes, Cynthia L. Stokes, David L. Trumper, Rebecca L. Carrier, Murat Cirit, Linda G. Griffith, Douglas A. Lauffenburger

**Affiliations:** ^1^ Department of Biological Engineering Massachusetts Institute of Technology 77 Massachusetts Avenue, Cambridge Massachusetts, 02139; ^2^ CN Bio Innovations Welwyn Garden City Hertfordshire UK; ^3^ Department of Pharmaceutical Sciences School of Pharmacy University of Pittsburgh Pittsburgh Pennsylvania; ^4^ Stokes Consulting Redwood City California; ^5^ Department of Mechanical Engineering Massachusetts Institute of Technology Cambridge Massachusetts; ^6^ Department of Chemical Engineering Northeastern University Boston Massachusetts; ^7^ Department of Chemical Engineering Massachusetts Institute of Technology Cambridge Massachusetts

**Keywords:** microphysiological system, organ‐on‐a‐chip, gut‐liver interaction, sepsis, CXCR3 ligands

## Abstract

A capability for analyzing complex cellular communication among tissues is important in drug discovery and development, and in vitro technologies for doing so are required for human applications. A prominent instance is communication between the gut and the liver, whereby perturbations of one tissue can influence behavior of the other. Here, we present a study on human gut‐liver tissue interactions under normal and inflammatory contexts, via an integrative multi‐organ platform comprising human liver (hepatocytes and Kupffer cells), and intestinal (enterocytes, goblet cells, and dendritic cells) models. Our results demonstrated long‐term (>2 weeks) maintenance of intestinal (e.g., barrier integrity) and hepatic (e.g., albumin) functions in baseline interaction. Gene expression data comparing liver in interaction with gut, versus isolation, revealed modulation of bile acid metabolism. Intestinal FGF19 secretion and associated inhibition of hepatic CYP7A1 expression provided evidence of physiologically relevant gut‐liver crosstalk. Moreover, significant non‐linear modulation of cytokine responses was observed under inflammatory gut‐liver interaction; for example, production of CXCR3 ligands (CXCL9,10,11) was synergistically enhanced. RNA‐seq analysis revealed significant upregulation of IFNα/β/γ signaling during inflammatory gut‐liver crosstalk, with these pathways implicated in the synergistic CXCR3 chemokine production. Exacerbated inflammatory response in gut‐liver interaction also negatively affected tissue‐specific functions (e.g., liver metabolism). These findings illustrate how an integrated multi‐tissue platform can generate insights useful for understanding complex pathophysiological processes such as inflammatory organ crosstalk. Biotechnol. Bioeng. 2017;114: 2648–2659. © 2017 Wiley Periodicals, Inc.

## Introduction

In drug discovery and development, animal models have been valuable for dissecting some biological mechanisms, but they are inadequate for recapitulating polygenic and multifactorial human diseases with diverse clinical phenotypes. As a result, drug attrition in Phase II and III clinical trials is now attributed mainly to a lack of clinical efficacy rather than toxicity (Cook et al., 2014; Denayer et al., 2014; Kubinyi, 2003). The problem of providing better biological models for human disease as is needed for efficacy studies, together with challenges remaining in certain more complex drug toxicity mechanisms, strongly motivates development of increasingly sophisticated in vitro models of human tissues and organs, so‐called “microphysiological systems (MPSs),” employing 3D tissue engineering approaches and often combined with microfluidics to control nutrient and drug distribution and mechanical stimulation (Domansky et al., 2010; Huebsch et al., 2016; Huh et al., 2012; Jeon et al., 2015; Mathur et al., 2015; Roth and Singer, 2014; Sobrino et al., 2016; Sung et al., 2013; Wikswo, 2014; Zhu et al., 2016).

Seminal work by Shuler and co‐workers demonstrated that aspects of preclinical pharmacokinetics (PK), pharmacodynamics (PD), and toxicity involving multiple different organs and tissues could be captured in vitro by connecting MPSs representing liver, fat, lung, and other organ mimics together using microfluidics, along with mathematical models to guide design, operation, and interpretation of the experimental systems (Sin et al., 2004; Sung and Shuler, 2009; Sung et al., 2010; Viravaidya et al., 2004). This early work stimulated development of a variety of new multi‐MPS platforms aimed at illuminating multi‐organ interactions in pharmacological applications (Esch et al., 2014; Loskill et al., 2015; Maschmeyer et al., 2015b; Materne et al., 2015; Miller and Shuler, 2016; Oleaga et al., 2016; Rebelo et al., 2016; Sin et al., 2004; Sung and Shuler, 2009; Sung et al., 2010; Viravaidya et al., 2004), with some of these moving into commercial application for important pre‐clinical assays.

Here, we extend the concept of multi‐MPS platforms to examine inflammation‐associated pathophysiology, as a step toward modeling complex human disease states in vitro in ways that may be useful in drug discovery. This work is motivated by recent advances in systems biology, where cell‐cell communication within and across multiple organs is conceptualized in the form of networks and modeled accordingly to address pathophysiology effectively (Gustafsson et al., 2014).

We focus here on gut‐liver interactions, as gut‐liver crosstalk is an integral part of normal physiology and its dysregulation is a common denominator in many disease conditions (Marshall, 1993). Gut and liver are major organs involved in drug absorption and metabolism, and changes to their functional interactions, such as those precipitated by injury or disease, can impact their responses to therapeutic intervention (Deng et al., 2009; Long et al., 2016; Morgan, 2001). The liver receives most of its blood supply from the gut via the portal circulation so it is constantly exposed to gut‐derived factors, including metabolites, microbial antigens, and inflammatory mediators. The gut‐liver axis contributes considerably to the overall immunological state of the body, with the gut being the largest immune organ and the liver harboring over 70% of the total macrophage population in the body (Marshall, 1993). It is well appreciated that interspecies differences often hinder the accurate prediction of human responses from animal models, and discrepancy is especially evident in processes involving the immune system (Denayer et al., 2014; Giese and Marx, 2014; Mestas and Hughes, 2004). Therefore, a fundamental understanding of gut‐liver crosstalk is critical not only to the prediction of drug disposition, efficacy, and toxicity, but also the elucidation of pathophysiological mechanisms.

In this study, we present an integrative molecular characterization of gut‐liver interactions in a dual‐organ MPS using a battery of continuous and endpoint metrics including clinically‐relevant biomarkers along with medium cytokine/chemokine assays and tissue mRNA profiling, under baseline and inflammatory conditions. Although our MPS constructs may be seen as relatively simple, comprising epithelial and immune cells but lacking a number of stromal cell types of each organ, the results recapitulate known crosstalk between gut and liver under baseline conditions as well as identify novel synergies in systemic inflammatory contexts. Inflammatory gut‐liver communication was associated with extensive modifications to hepatic biotransformation and detoxification pathways (e.g., cytochrome P450), which are key determinants of drug responses. This approach, integrating multi‐tissue MPS systems and quantitative analysis of inflammation, is generalizable to study more complex MPS constructs and higher order (>2) organ interactions, thus potentially has broad applicability toward advancing fundamental understanding of human (patho)physiology and drug development.

## Materials and Methods

### Liver MPS Preparation

The liver MPS was prepared as previously described (Long et al., 2016). Human primary hepatocytes and Kupffer cells were purchased from Life Technologies (HMCPMS, HUKCCS, Waltham, MA). The liver scaffold was a thin (0.25 mm) polystyrene disc perforated with 301 channels (diameter = 0.3 mm) (Clark et al., 2016). Scaffolds were washed in 70% EtOH for 15 min, rinsed twice in PBS, coated with 30 μg/mL rat tail collagen in PBS for 1 h at room temperature. The collagen‐coated scaffolds were air‐dried, and punched into platforms. At day(‐3) to experiment start, hepatocytes, and Kupffer cells were thawed in Cryopreserved Hepatocyte Recovery Medium (CHRM, Invitrogen, Waltham, MA), spun down at 100 × g for 8 min, and seeded at 10:1 ratio, 6 × 10^5^:6 × 10^4^ cells/well, in hepatocyte seeding medium (250 mL Advanced DMEM, 9 mL Gibco Cocktail A, 12.5 mL fetal bovine serum [FBS]). After 1 day, the media was changed to D(‐2) medium (250 mL Advanced DMEM, 10 mL Cocktail B).

### Gut MPS Preparation

Caco2 (clone: C2BBe1, passage 48–58, ATCC, Manassas, VA) and HT29‐MTX (passage 20–30, Sigma–Aldrich, St. Louis, MO) were maintained in DMEM (Gibco, Gaithersburg, MD) supplemented with 10% heat‐inactivated FBS (Atlanta Biologicals, Flowery Branch, GA), 1% GlutaMax (Gibco), 1% Non‐Essential Amino Acids (NEAA, Gibco), and 1% Penicillin/Streptomycin (P/S). Both cell lines were passaged twice post thawing before their use for transwell seeding. Briefly, the apical and basal side of transwell membrane were coated with 50 μg/mL Collagen Type I (Corning Inc., Corning, NY) overnight at 4°C. Caco2 at ∼70–80% confluence and HT29‐MTX at ∼80–90% confluence were harvested using 0.25% Trypsin‐EDTA and mechanically broken up into single cells. 9:1 ratio of C2BBe1 to HT29‐MTX was seeded onto 12‐well 0.4 μm pore polyester transwell inserts (Corning, Tewksbury, MA) at a density of 10^5^ cells/cm^2^. Seeding media contained 10% heat‐inactivated FBS, 1x GlutaMax and 1% P/S in Advanced DMEM (Gibco). Seven days post seeding, the media was switched to a serum‐free gut medium by replacing FBS with Insulin‐Transferrin‐Sodium Selenite (ITS, Roche, Indianapolis, IN) and the epithelial cultures were matured for another 2 weeks.

Monocyte‐derived dendritic cells were used as the immune component of the gut MPS. Briefly, peripheral blood mononuclear cells (PBMCs) were processed from Leukopak (STEMCELL Technologies, Vancouver, BC, Canada). Monocytes were isolated from PBMCs using the EasySep Human Monocyte Enrichment Kit (STEMCELL Technologies, 19058) and were differentiated in Advanced RPMI medium (Gibco) supplemented with 1x GlutaMax, 1% P/S, 50 ng/mL GM‐CSF (Biolegend, San Diego, CA), 35 ng/mL IL4 (Biolegend), and 10 nM Retinoic acid (Sigma). After 7 days of differentiation (at day 19–20 post epithelial cell seeding), immature dendritic cells were harvested using Accutase (Gibco) and seeded onto the basal side of the gut transwells. One‐day post dendritic cell seeding, gut barrier function was assessed. Gut MPS with transepithelial electrical resistance (TEER) values of at least 250 Ohm × cm^2^ were considered acceptable for experiment.

For all interaction experiments, the gut MPS was maintained in serum‐free apical medium consisting of Phenol red‐free DMEM (Gibco) supplemented with 1x ITS, 1% NEAA, 1% GlutaMax and 1% P/S. Gut basal and all compartments that were fluidically linked to systemic circulation were fed with serum‐free common media, which contained 500 mL William's E medium, 20 mL Gibco Cocktail B, 100 nM hydrocortisone, and 1% P/S.

Detailed Materials and Methods are in Supporting Information.

## Results

### Overview of Study Design

**Figure 1:**
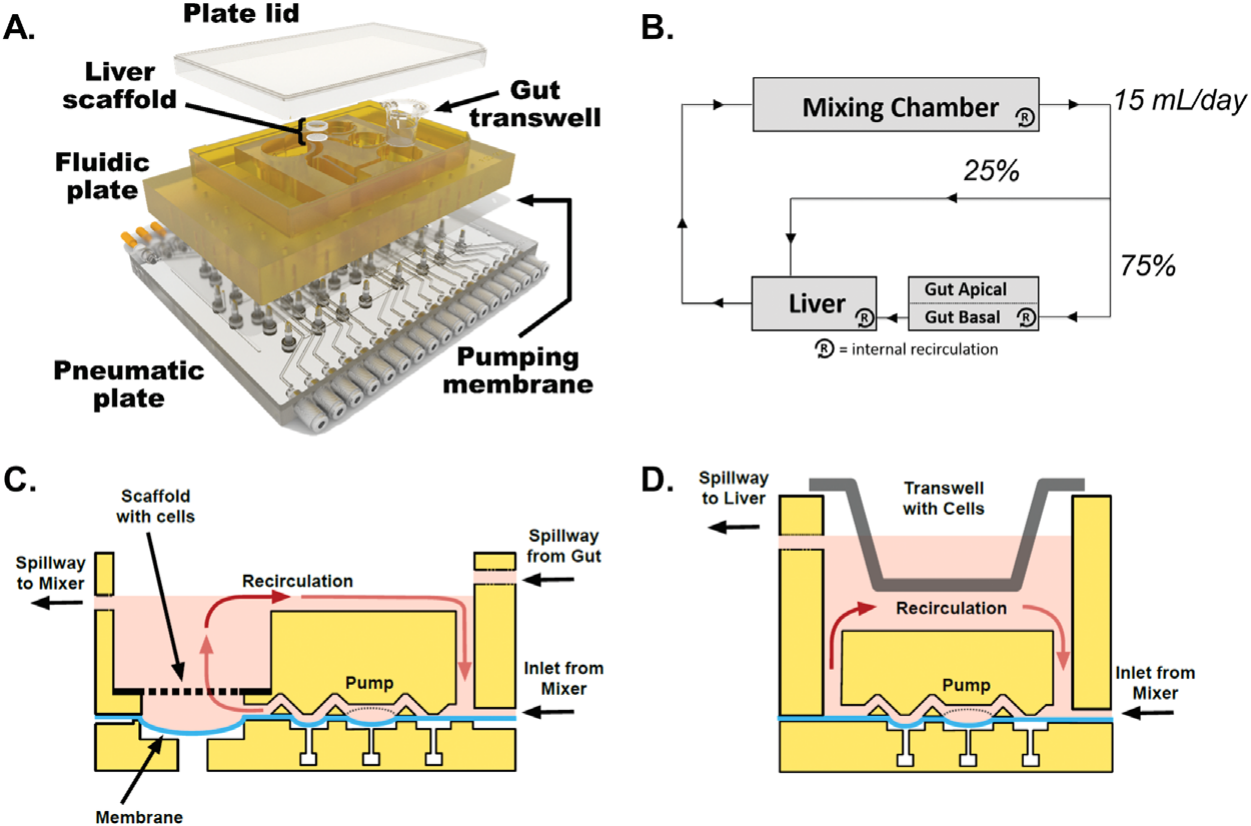
Overview of platform design and operation. (**A**) Exploded view of a multi‐MPS platform. Top plate (shown in yellow polysulfone) contains MPS compartments and distributes culture medium through micromachined channels and pumps on its bottom face. Bottom plate (shown in clear acrylic) distributes compressed air and vacuum to small ports below each pump/valve chamber. A membrane layer (translucent polyurethane) clamped between the two plates seals the channels and provides a sterile barrier while serving as the actuation layer of the pumps and valves. Stainless steel screws fasten the layers together into a single unit that can be handled like a traditional well plate. (**B**) A flow partitioning schematic illustrates the gut‐liver communication circuit and intra‐MPS mixing within each compartment. (**C**) Schematic of the perfused liver MPS on platform (not to scale). The liver module contains a rigid, perfused polystyrene scaffold with 301 microchannels that serve to localize and aggregate primary human hepatocytes and Kupffer cells into miniature liver tissues. A recirculation pump (1 μL/s) maintains continuous flow across a region of shallow geometry to provide efficient oxygenation, while a suspended portion of the pumping membrane serves as a capacitor to smooth the pressure profile of the peristaltic pump. The module integrates fluid inputs from the mixing chamber and gut compartment, while shunting excess volume back to the mixer via an outflow spillway. (**D**) Schematic of the perfused gut MPS on platform (not to scale). A recirculation pump (0.25 μL/s) maintains continuous flow across basal side of the gut MPS to provide continuous mixing. The module receives fluid input from the mixing chamber while shunting excess volume to the liver compartment via an outflow spillway.

We designed and implemented a multi‐MPS platform hosting immune‐competent gut and liver models to enable quantitative analysis of the gut‐liver interactome, as illustrated in Figure 1.

### Hardware Design and Operation

The ability to derive useful data from MPS platforms for in vitro–in vivo translation depends on choosing hardware designs that permit quantitative analysis of relevant phenomena, including drug fate and exposure. While most OOC systems are fabricated from polydimethylsiloxane (PDMS)—a versatile elastomer that is easy to prototype, has good optical properties, and is oxygen permeable—hydrophobic compounds, including steroid hormones and many drugs, strongly partition into PDMS, thus precluding quantitative analysis and control of drug exposures (Toepke and Beebe, 2006). We therefore use a platform fabricated from polysulfone via micromachining, with on‐board pneumatic microfluidic pumping (Domansky et al., 2010; Inman et al., 2007) adapted from technology now used commercially in the Liverchip™ for extended 3D culture of functional liver tissue (Long et al., 2016; Sarkar et al., 2017, 2015; Tsamandouras et al., 2017; Vivares et al., 2015), and for demonstration of human liver CYP450 regulation by inflammation (Long et al., 2016). This on‐board pumping technology minimizes space, auxiliary equipment, and dead volumes associated with excess tubing (Domansky et al., 2010; Inman et al., 2007).

Multi‐MPS platforms can employ a variety of pumping schemes including gravity flow, external flow, and on‐board low‐capacity pumps providing either recirculation or once‐through perfusion of tissues or cell monolayers (Esch et al., 2014, 2016; Frey et al., 2014; Iori et al., 2012; Lee et al., 2017; Loskill et al., 2015; Maschmeyer et al., 2015a; Miller and Shuler, 2016; Oleaga et al., 2016). A distinctive feature of our platform is the use of separate on‐board, high‐capacity, pulse‐damped pumps (Domansky et al., 2010; Inman et al., 2007) to circulate medium within each individual MPS compartment, as well as between the MPSs. This advantage is crucial, as the within‐MPS recirculation enables micro‐perfusion through the 3D liver tissue that provides adequate oxygenation and distribution of nutrients through the entire tissue, with an independent oxygenation loop that does not depend on between‐MPS fluid exchange (Domansky et al., 2010; Inman et al., 2007; Powers et al., 2002a,2002b). It concomitantly provides internal high‐flow recirculation on the basal side of the transwell to enhance mass transfer. This uncoupling of internal MPS mass transfer from external MPS–MPS communication offers a feature especially important for 3D MPS configurations with high metabolic demands or need for high rates of internal mass transfer within the MPS. The liver MPS used in the present work was designed with consideration of transport and reaction of nutrients and drugs, with experimental measurements of oxygen and distribution of gene therapy vectors and other agents compared to design models as previously described (Domansky et al., 2010; Powers et al., 2002a,2002b). The flow rate used for internal recirculation in the liver is more than fivefold greater than the flow rate for exchange of medium between the liver MPS and the system; hence, addition of this exchange flow should exert negligible influence on the oxygenation status of the liver, or on the mass transfer in the liver tissue.

The platform has a footprint similar to a typical multi‐well plate with chambers designed to house different types of microtissues as shown in Figure 1A. It can be reconfigured to accommodate 2‐, 3‐, and 4‐way interactions, with user‐defined control of flow rates and flow partitioning from the mixing chamber to the different tissue compartments. Precise flow rate control can be achieved over a wide range (0–432 mL/day, 0.5 uL per stroke, at frequencies up to 8 Hz).

The fluidic communication facilitating gut‐liver MPS interactions here was modeled based on physiological flows. In vivo, the liver receives a dual blood supply, from the hepatic artery and the portal vein (Brown et al., 1997; Liaskou et al., 2012). Correspondingly, we specified the flow partitioning from mixer into the gut and liver compartment to be 75% and 25%, respectively (Fig. 1B) (Brown et al., 1997). The output from the gut module fed into the liver, representing portal circulation. A systemic (i.e., global between‐MPS) flow rate of 15 mL/day was used and individual MPS recirculation rates were set at values to provide adequate nutrient and drug exchange; specifically, 1 μL/s for liver and 0.25 μL/s for gut. The total volume of medium in the liver compartment was 1.6 mL, in the gut compartment 0.50 mL apical and 1.5 mL basal, and in the mixing chamber 1.0 mL. A summary of the operational parameters is provided in Supporting Information (Tables SI and SII).

### Development and Scaling of Multi‐Cellular Gut and Liver MPS

Multicellular and (innate) immune‐competent gut and liver tissue constructs were used, to encompass interacting metabolic, barrier, and immune functions. The liver microtissue comprised a co‐culture of human primary cryopreserved hepatocytes and Kupffer cells at physiological 10:1 ratio (Ebrahimkhani et al., 2014), maintained in a culture medium permissive for retention of inflammation responses by adjustment of cortisol within a physiological rather than supraphysiological range (Long et al., 2016; Sarkar et al., 2015). It has been previously observed that Kupffer cells can survive interspersed among hepatocytes for 2‐weeks in a similar liver bioreactor system (Wheeler et al., 2014). Although some investigators have employed a more diverse population of primary human non‐parenchymal liver cells in co‐culture with hepatocytes (Esch et al., 2015, 2016; Feaver et al., 2016), these are not as reliably available in cryopreserved form, so rather than using cell lines (Esch et al., 2014; Oleaga et al., 2016), or stellate cells activated by culture before cryopreservation, we employed a minimal model using available cryopreserved cells that had not been in culture prior to cryopreservation. The gut tissue was engineered to recapitulate key aspects of the small intestine (Noah et al., 2011), with the epithelial monolayer derived from 9:1 ratio of absorptive enterocytes (Caco2‐BBE) and mucus‐producing goblet cells (HT29‐MTX), co‐cultured with primary monocyte‐derived dendritic cells.

Diverse approaches to scaling in MPS systems have been pursued (Abaci and Shuler, 2015; Maass et al., 2017; Moraes et al., 2013; Sung et al., 2010; Wikswo et al., 2013), and many variables including ratios of particular cell types, cell‐medium ratios, flow rates, residence times within organs, recirculation versus once‐through modes, and others that all address how to translate data from a minimal system to a prediction of human in vivo response. As our present inflammation biology‐focused study was carried out in parallel with a companion detailed analysis investigating drug PK in the same system (manuscript submitted), where the absorptive and metabolic capacities of the gut and liver are a key function of interest, the gut surface area to hepatocyte number ratio was chosen as a key scaling factor. The ratio of small intestine epithelium surface area (1.12 cm^2^ in the gut MPS, 30 m^2^ in humans, Helander and Fandriks, 2014) to hepatocytes (600,000 in the liver MPS, 3 × 10^11^ in humans, Prothero, 1982) on the gut‐liver platform is on the same order of magnitude as in vivo (1.86 × 10^−10^ in vitro and 1.00 × 10^−10^ in vivo).

Features of the innate immune system were considered in scaling the liver, with the number of Kupffer cells (which comprise the majority of innate immune cells in liver) to hepatocytes (1:10) similar to in vivo (Ebrahimkhani et al., 2014). The gut also contains an element of the innate immune system, dendritic cells. However, the relative number of dendritic cells to epithelial cells in the human gut is not well‐characterized. Our gut MPS contains approximately 1:10 dendritic cells (1.0 × 10^5^ cells) to intestinal epithelial cells (monolayer contains ∼1.0 × 10^6^ cells at maturation), similar to the immune/epithelial cell ratio in the liver. Additional considerations in scaling the in vivo–in vitro physiology of this gut‐liver interaction (e.g., the cell‐to‐medium ratio) are described in the Discussion.

### Long‐Term Maintenance of Baseline Liver‐ and Gut‐Specific Functions in Gut‐Liver Interactome

**Figure 2:**
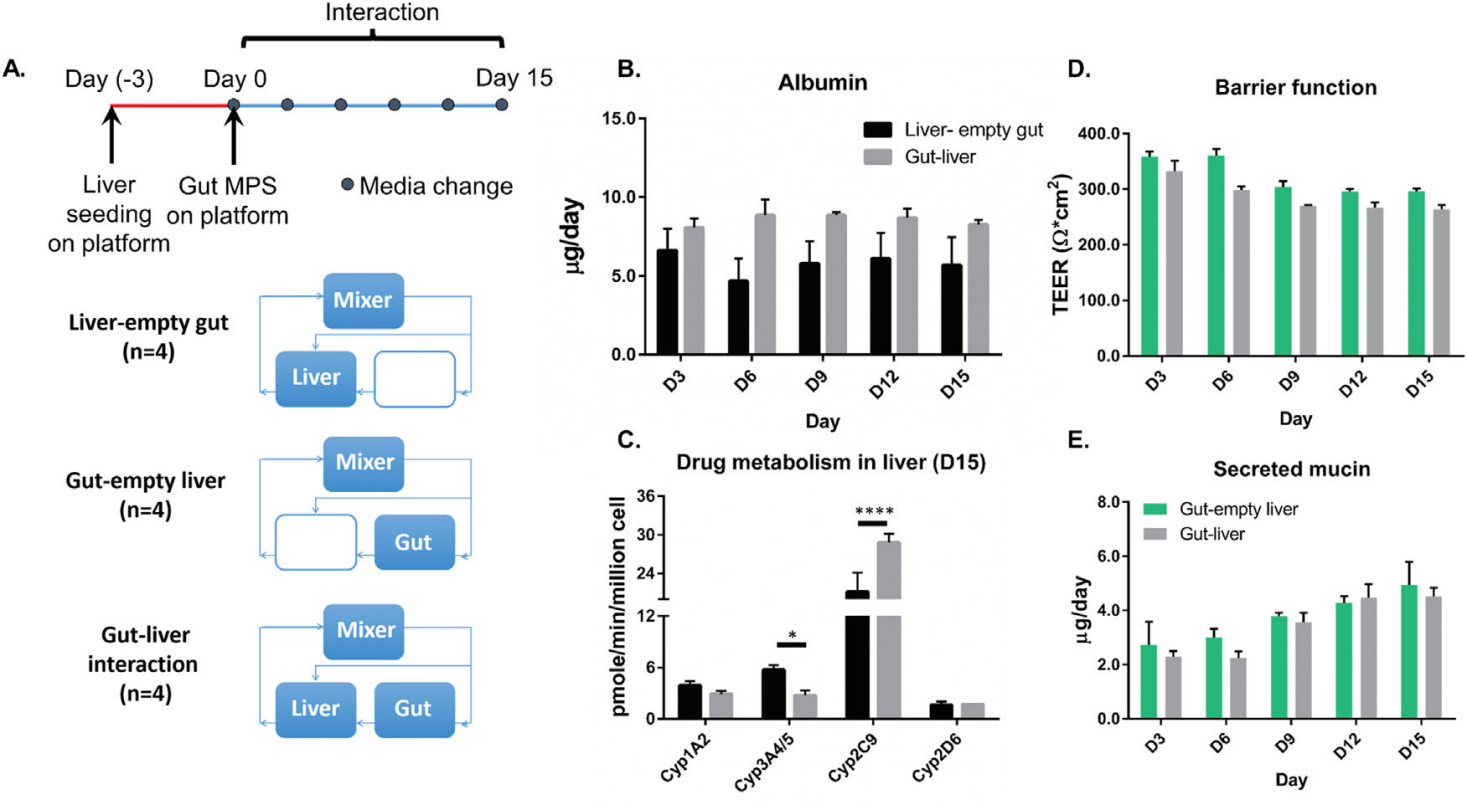
(**A**) Overview of experimental timeline and design. Media was replaced every 3 days for the duration of the 2‐week interaction. (**B**) Long‐term maintenance of liver‐specific albumin production. (**C**) Evaluation of hepatic drug metabolic enzyme activities after 15 days of gut‐liver interaction. *P ≤ 0.05 and ****P < 0.0001 denote statistical significance after Holm‐Sidak multiple‐comparison correction. (**D**) Two‐week maintenance of intestinal barrier function. (**E**) Two‐week maintenance of intestinal mucin production. *N* = 3‐4, mean ± SD.

To evaluate long‐term functional viability in the gut‐liver interaction, we included corresponding single tissue controls on platform, operated with identical media volumes, flow rates, and flow partitioning (Fig. 2A). All conditions were tested in a defined, serum‐free common media that supported maintenance of both gut and liver functions. The liver cells were seeded on platform 3 days prior to the start of the interaction experiment to allow for tissue formation and recovery from seeding‐related stress responses. The gut MPS was differentiated for 3 weeks off‐platform prior to the start of the interaction experiment. During long‐term operation, the common culture medium in the system was replaced every 3 days for comparison to historical performance of the liver MPS and with prior knowledge that basal nutrients such as glucose are not depleted during this time, along with a desire to allow interactions to evolve dynamically unperturbed; an alternate scheme of partial daily medium exchange is the subject of future investigation.

Hepatic and intestinal functions assessed over 2 weeks of culture were comparable for MPS maintained in communication or in isolation, as assessed by measurements of albumin production, gut barrier integrity, and gut mucus production (Fig. 2). The albumin production rate (Fig. 2B) obtained from the interaction platform was in agreement with levels previously reported in human hepatocyte cultures across multiple donors (Tsamandouras et al., 2017). The gut barrier function (Fig. 2D), as indicated by TEER measurement, was also comparable to previously reported values for Caco2:HT29‐MXT co‐cultures (Mahler et al., 2012; Nollevaux et al., 2006) as well as primary intestinal monolayers (VanDussen et al., 2015). To evaluate liver metabolic function at the end of the 2‐week experiment, the liver tissues from isolation and interaction conditions (in the absence of gut) were dosed with a cocktail of drug substrates targeting specific CYP450 enzymes. Drug‐specific metabolite production in the media was measured using mass spectrometry to determine the cytochrome P450 activity of the different isoforms. Overall, the liver metabolic function was largely maintained, with modulation of select cytochrome P450 activities observed in gut‐liver interaction (Fig. 2C). In particular, Cyp2C9 activity was significantly enhanced, while Cyp3A4/5 activity was depressed.

### Modulation of Bile Acid Synthesis Pathway in Gut‐Liver Crosstalk

**Figure 3:**
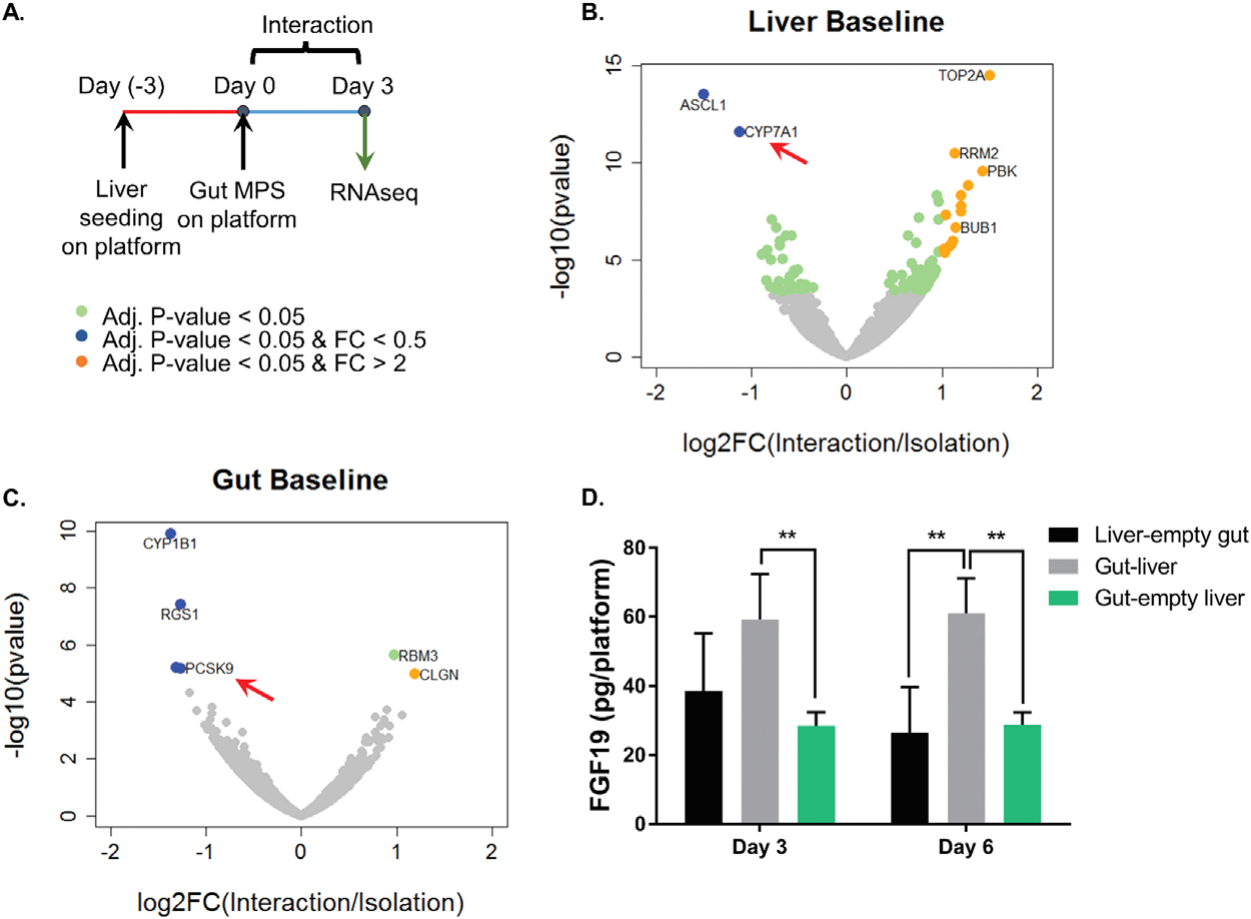
(**A**) Overview of experimental design. (**B**) Volcano plot illustrates the differentially expressed genes in the liver under baseline interaction versus isolation. Select biological processes up‐ and down‐regulated based on the differentially expressed gene (Adj. *P*‐values < 0.05) in 3B are displayed in Tables I and II. (**C**) Volcano plot illustrates the differentially expressed genes in the gut under baseline interaction versus isolation. Data point in green indicate statistically differentially expressed genes (FDR‐adj. *P*‐values < 0.05). Points in orange indicate significantly upregulated genes with effect size greater than twofold. Points in blue indicate significantly down‐regulated genes effect size greater than twofold. (**D**) FGF19 secretion was measured using ELISA. **P ≤ 0.01 denote statistical significance after Holm‐Sidak multiple‐comparison correction. *N* = 4, mean ± SD.

While the standard phenotypic and functional metrics were not extensively altered in baseline gut‐liver interaction (Fig. 2), these tissues interact in myriad ways beyond the protein‐level metrics examined. In order to reveal potential modulation of cell regulatory processes, we performed RNA sequencing to profile the global transcriptomic changes in the gut and liver tissues after 3 days of interaction, with corresponding isolation controls (i.e., gut‐only and liver‐only). A total of 105 genes were significantly (FDR‐adjusted *P* < 0.05) altered in the liver during interaction relative to isolation controls, of which 70 were upregulated and 35 were downregulated (Fig. 3B). For the gut, six genes were significantly differentially expressed, of which two were upregulated and four were downregulated (Fig. 3C). To understand the functional implications of these molecular changes, we performed Gene Ontology (GO) analysis to identify overrepresented biological processes that were altered under interaction. Only significantly altered genes (FDR‐adjusted *P *< 0.05) were used for GO analysis. The biological processes up‐regulated in the liver during baseline gut‐liver interaction involved processes in cell division (Table I). Induction of cell cycle genes in the liver may indicate an adaptive response to gut‐derived signals, although the soluble factors involved are unknown. On the other hand, the biological processes down‐regulated in the liver in interaction were characterized by metabolic processes, including bile acid biosynthesis and xenobiotic metabolism (Table II). Of particular interest is the regulation of the bile acid metabolism mediated by CYP7A1. CYP7A1 is an enzyme central to bile acid synthesis, and its inhibition by FGF19 in enterohepatic communication is well established (Ding et al., 2015). Suppression of CYP7A1, therefore, indicates a physiologically known coupling of gut‐liver functions. We measured FGF19 production in gut‐liver interaction and corresponding isolation controls to show that FGF19, albeit at low concentration overall, was indeed higher in the interacting system (Fig. 3D). Therefore, the suppression of hepatic CYP7A1 expression could be attributed to intestinal FGF19 signaling. Although the number of significant genes in the gut samples was insufficient for GO analysis, it is intriguing to note that PCSK9, one of the differentially expressed genes, plays a key role in cholesterol and lipid homeostasis. In fact, cholesterol and various types of bile acids have been shown to suppresses PCSK9 mRNA expression in Caco2 intestinal cultures (Leblond et al., 2009).

**Table I:** Select biological processes up‐regulated in liver under gut‐liver interaction

GO ID	Biological processes	*P*‐value^a^	Adj. *P*‐value^a^
GO:0051302	Regulation of cell division	0.0E+00	0.0E+00
GO:0000070	Mitotic sister chromatid segregation	0.0E+00	0.0E+00
GO:0007059	Chromosome segregation	0.0E+ 00	0.0E+00
GO:0007049	Cell cycle	9.6E−18	1.1E−14
GO:0006996	Organelle organization	7.3E−10	3.6E−07
GO:0008283	Cell proliferation	3.4E−09	1.4E−06
GO:0007017	Microtubule‐based process	4.9E−08	1.4E−05

^a^ *P*‐values below the GOseq threshold are denoted as 0.0E+0.0.

**Table II:** Select biological processes down‐regulated in liver under baseline gut‐liver interaction

GO ID	Biological processes	*P*‐value	Adj. *P*‐value
GO:0006694	Steroid biosynthetic process	2.2E−05	1.5E−01
GO:0006579	Amino‐acid betaine catabolic process	2.8E−05	1.5E−01
GO:0008202	Steroid metabolic process	4.7E−05	1.5E−01
GO:0006699	Bile acid biosynthetic process	4.1E−05	1.5E−01
GO:1901617	Organic hydroxy compound biosynthetic process	1.0E−04	2.6E−01
GO:0044283	Small molecule biosynthetic process	1.8E−04	3.8E−01
GO:0015914	Phospholipid transport	2.2E−04	3.9E−01
GO:0044281	Small molecule metabolic process	3.3E−04	4.8E−01
GO:0006805	Xenobiotic metabolic process	5.2E−04	5.5E−01

While the liver MPS used here does not have a separate biliary drainage to the luminal part of the gut, bile is nonetheless produced by the hepatocytes and released into the MPS medium. We have previously demonstrated the production of the major bile acids (glycocholic acid, taurocholic acid, and cholic acid) in the Liverchip bioreactor (liver alone) under conditions similar to those used here (Sarkar et al., 2017). Circulating bile acid in the system can interact with the basal side of the gut; hence, even without apical stimulation of bile acids, some of the regulatory effects may be exerted through basal stimulation. Collectively, the convergence on cholesterol and bile acid metabolism pathways suggests transcriptional rewiring due to inter‐MPS communication, even if the communication captures only partial representation of the in vivo physiology.

### Coordinated Modulation of Gut and Liver Transcriptome in Inflammatory Gut‐Liver Crosstalk

**Figure 4:**
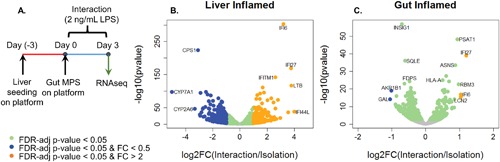
(**A**) Overview of experimental design. (**B**) Volcano plot illustrates the differentially expressed genes in the inflamed liver under interaction versus isolation. Select biological processes up‐ or down‐regulated based on the differentially expressed gene (Adj. *P*‐values < 0.05) in **B** are displayed in Tables III and IV. (**C**) Volcano plot illustrates the differentially expressed genes in the inflamed gut under interaction versus isolation. Biological processes up‐ or down‐regulated based on the differentially expressed gene (Adj. *P*‐values < 0.05) in **C** are displayed in Tables V and VI. *N* = 4, mean ± SD.

After demonstrating homeostatic gut‐liver crosstalk in the baseline condition, we investigated gut‐liver interplay in simulated endotoxemia, a condition characterized by increased concentrations of circulating lipopolysaccharide (LPS). Endotoxemia is observed in a spectrum of clinical presentations, with low levels (1–2 ng/mL) causing mild inflammation and high concentrations (2–10 ng/mL) associated with severe inflammation (Guo et al., 2013). The design of the experiment was analogous to the baseline study, comprising interaction and isolation controls, with the only difference being the addition of 2 ng/mL LPS in the circulating media (Fig. 4A).

RNA‐seq was performed to assess the global molecular changes associated with inflammatory gut‐liver crosstalk. For the liver, 2548 genes were significantly altered in the interaction, of which 1137 genes were upregulated and 1411 genes were downregulated (Fig. 4B). GO analysis of the differentially expressed genes indicated upregulation of pathways involved in immune response and interferon signaling and downregulation of pathways involved in lipid and xenobiotic metabolism (Tables III and IV). For the gut, 780 genes were significantly altered during interaction, of which 290 genes were upregulated and 490 genes were downregulated (Fig. 4C). Similarly, GO analysis revealed upregulation of defense response and interferon signaling; down‐regulated pathways included alcohol biosynthesis, steroid and lipid metabolism (Tables V and VI). In addition to gene‐based GO analysis, we also performed Gene Set Enrichment Analysis (GSEA) on the RNAseq data, which yielded similar conclusions (Supporting Information Fig. S1).

**Table III:** Select biological processes up‐regulated in liver under inflammatory gut‐liver interaction

GO ID	Biological processes	*P*‐value	Adj. *P*‐value
GO:0006955	Immune response	1.7E−28	2.3E−24
GO:0006952	Defense response	1.5E−27	1.0E−23
GO:0019221	Cytokine‐mediated signaling pathway	2.5E−25	4.6E−22
GO:0060337	Type I interferon signaling pathway	2.0E−25	4.4E−22
GO:0051707	Response to other organism	8.8E−22	7.6E−19
GO:0034341	Response to interferon‐gamma	1.7E−22	1.8E−19

**Table IV:** Select biological processes down‐regulated in liver under inflammatory gut‐liver interaction

GO ID	Biological processes	*P*‐value	Adj. *P*‐value
GO:0044281	Small molecule metabolic process	7.8E−97	1.0E−92
GO:0006082	Organic acid metabolic process	5.4E−78	1.8E−74
GO:0055114	Oxidation‐reduction process	5.4E−69	1.4E−65
GO:0044710	Single‐organism metabolic process	2.3E−56	5.0E−53
GO:0032787	Monocarboxylic acid metabolic process	4.0E−54	7.4E−51
GO:0006629	Lipid metabolic process	7.4E−53	9.7E−50
GO:0006805	Xenobiotic metabolic process	1.3E−23	5.7E−21

**Table V:** Select biological processes up‐regulated in gut under inflammatory gut‐liver interaction

GO ID	Biological processes	*P*‐value	Adj. *P*‐value
GO:0006952	Defense response	4.5E−20	5.3E−16
GO:0060337	Type I interferon signaling pathway	1.2E−19	5.3E−16
GO:0002376	Immune system process	1.4E−13	2.2E−10
GO:0034097	Response to cytokine	9.2E−13	1.1E−09
GO:0034341	Response to interferon‐gamma	1.2E−11	8.9E−09

**Table VI:** Select biological processes down‐regulated in gut under inflammatory gut‐liver interaction

GO ID	Biological processes	*P*‐value	Adj. *P*‐value
GO:0046165	Alcohol biosynthetic process	5.5E−16	7.0E−12
GO:0008202	Steroid metabolic process	2.6E−14	1.0E−10
GO:1901615	Organic hydroxy compound metabolic process	3.2E−14	1.0E−10
GO:0044281	Small molecule metabolic process	4.4E−12	4.3E−09
GO:0032787	Monocarboxylic acid metabolic process	4.8E−11	3.8E−08
GO:0006629	Lipid metabolic process	7.8E−11	5.9E−08
GO:0055114	Oxidation‐reduction process	5.0E−08	2.4E−05

Hepatic clearance of endogenous and xenobiotic compounds is mediated by two mechanisms: metabolism and bile elimination; inflammatory crosstalk negatively affected both of these processes. Collectively, CYP1A2, CYP2C9, CYP2C19, CYP2D6, CYP3A4, and 3A5 are responsible for the metabolism of over 90% of known drugs (Ebrahimkhani et al., 2014; Jacob et al., 2009); all of these were suppressed in the liver in the integrated system (Supporting Information Table SIII), likely due to accumulation of inflammatory mediators, such as IL6, TNFα, and type I interferons (Huang et al., 2010; Long et al., 2016).

### Synergies in Cytokine/Chemokine Production Under Inflammatory Gut‐Liver Crosstalk

**Figure 5:**
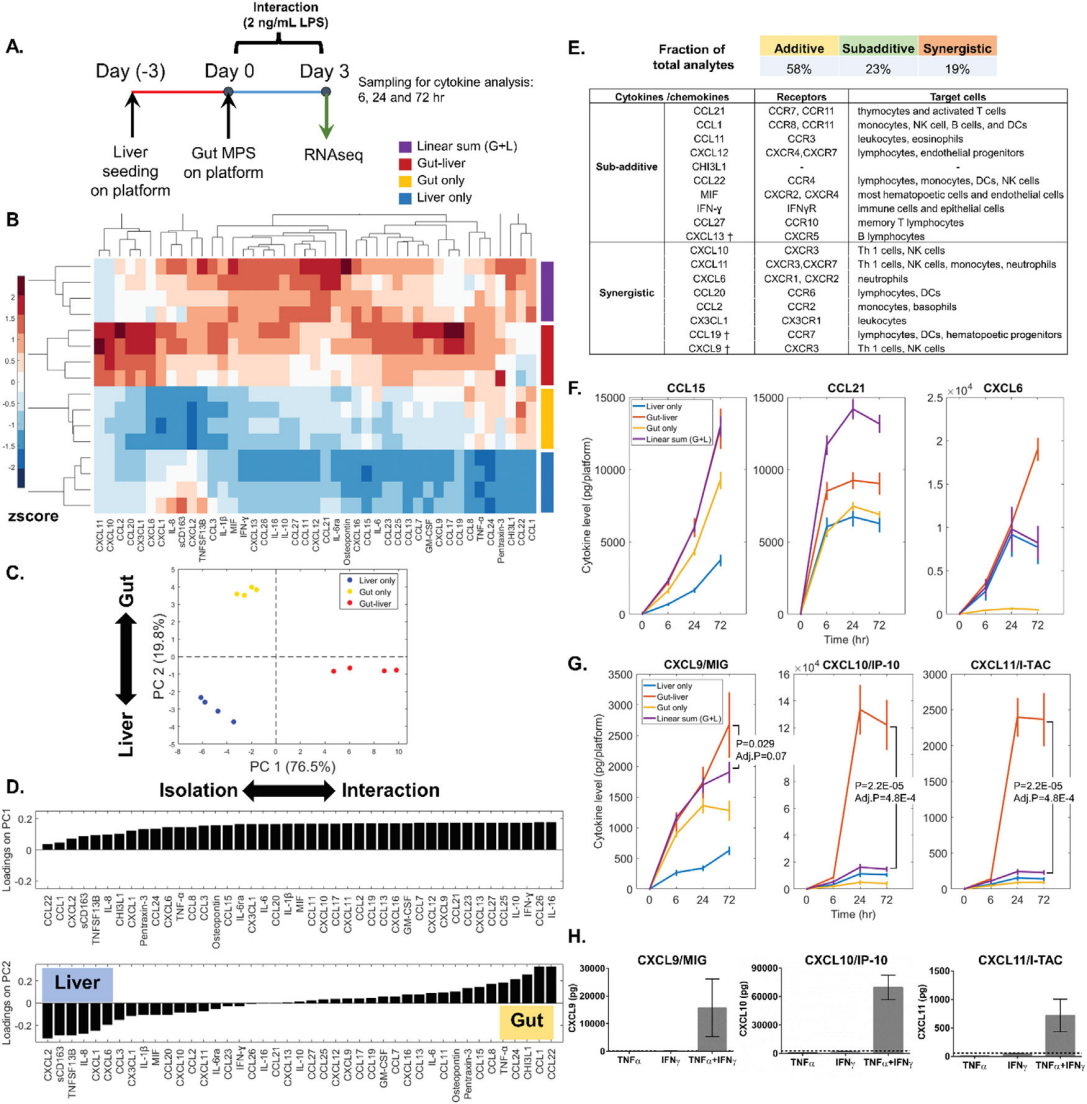
(**A**) Experimental outline. (**B**) Hierarchical clustering of 72‐h cytokine level in the different experimental conditions. The cytokine levels were volume normalized and the total amount of cytokine production per platform was obtained by summing the compartmental levels (gut + liver + mixer). Each analyte was mean centered and normalized by its standard deviation across all conditions. Color bar indicates the relative cytokine amount, with red and blue corresponds to high and low production, respectively. (**C**) Principal component analysis of the 72‐h cytokine profile. The score plot indicates that PC1 separated the conditions in isolation versus interaction; PC2 separated the conditions in gut and liver. (**D**) Loading plot displays the cytokines predominantly secreted by the liver or gut. (**E**) The list of cytokines/chemokines found to be statistically different from linear sum (Adj. *P*‐value < 0.05) and the corresponding receptors. † indicates borderline significance (0.05 < Adj. *P*‐value ≤ 0.07). (**F**) Illustration of different patterns of cytokine/chemokine regulation, including linearly additive, subadditive, and synergistic. (**G**) CXCR3 ligand production in the different experimental conditions. *N* = 4, mean ± SD. (**H**) TNFα (5 ng/mL) and IFNγ (5 ng/mL) synergistically enhanced CXCR3 ligand production in gut epithelial cells (24 h). The dash line in each plot indicates the theoretical chemokine level from summing the TNFα and IFNγ‐only conditions. *N* = 3, mean ± SD.

To examine temporal evolution of the inflammatory response, we measured secreted cytokines and chemokines in the media at 6, 24, 72 h post stimulation (Fig. 5A). Pairwise hierarchical clustering was performed on the 72 h cytokine measurement to explore the correlations of cytokine responses among the analytes and conditions (Fig. 5B). Unsupervised principal component analysis (PCA) revealed that the over 96% of the covariance in the cytokine dataset can be captured by the first two principal components. PC1 accounted for 76% of the variability in the data, segregating the interaction versus isolation controls; PC2 accounted for 20% of the total variability and discriminated the gut and liver only conditions (Fig. 5C). The loading plot depicts the relative contribution of each analyte to the 1st and 2nd principal components (Fig. 5D). While all analytes were positively loaded on PC1, reflecting increases in the interacting system, loadings on PC2 can aid in inferring the primary contributions of a tissues to the circulating cytokines/chemokines. While none of the soluble factors were unique to gut or liver, multivariate cytokine patterns can reveal tissue‐specific signatures.

In order to assess the importance of inter‐MPS crosstalk in the integrated inflammatory response, we compared the measured cytokine levels in the interacting system to the theoretical linear sum of the isolated conditions. Cytokine levels observed in isolation accounted for output due to direct TLR4 activation and intra‐MPS paracrine signaling. The actual (measured) cytokine levels in the integrated systems often deviated significantly from the linear sum of the isolated systems, revealing non‐linear modulation of cytokine production as a result of inter‐MPS communication (Fig. 5B). Although roughly 60% of the analytes were linearly additive, approximately 20% were less than additive and another approximately 20% were positively synergistic—some very markedly so (Figs. 5B,E,G and S2).

A notable synergistic amplification involved CXCR3 ligands, with CXCL10 (IP10) and CXCL11 (I‐TAC) being most significantly synergistic and CXCL9 (MIG) borderline significant (Fig. 5G). CXCR3 signaling has been implicated in myriad inflammatory diseases, including autoimmunity, inflammatory bowel disease (IBD), transplant rejection, infection, and cancer (Groom and Luster, 2011; Singh et al., 2007). Our results indicate that consideration of gut‐liver crosstalk is important for assessing systemic inflammatory processes and their potential contribution to disease development. RNA‐seq data implicated activation of IFNα/β/γ signaling pathways in both the gut and liver during organ crosstalk (Tables III and V and Supporting Information Fig. S1), and TNFα can magnify IFN‐dependent production of CXCR3 ligands (Groom and Luster, 2011). PCA loadings revealed that TNFα was predominately gut‐derived and IFNγ was produced at comparable levels by both the gut and the liver (Figs. 5D and S2). It is plausible that gut (dendritic cells)‐derived TNFα synergized here with tissue‐specific IFNγ signaling to drive enhanced CXCR3 ligand production in both the gut and liver.

Although immune cells are the principal responders to endotoxin due to higher expression of TLR4 (Supporting Information Table SX), epithelial cells also contribute to inflammation indirectly via activation by immune cell‐derived cytokines, such as TNFα and IL‐1 (Dwinell et al., 2001; Nguyen et al., 2015; Yeruva et al., 2008). To assess the epithelial contribution to the cytokine response, we stimulated the gut epithelium (Caco2‐BBE/HT29‐MTX) basally with 5 ng/mL TNFα, 5 ng/mL IFNγ, or both, for 24 h. Co‐treatment of TNFα and IFNγ on the gut epithelium, in the absence of immune cells, resulted in marked amplification of 4 of the 8 synergistic chemokines identified in the integrated system, including CXCL9, CXCL10, CXCL11 (Fig. 5H), and CX3CL1 (Supporting Information Fig. S3). These results corroborated with the RNA‐seq findings and demonstrated that IFNγ and TNFα signaling crosstalk was central to the synergistic chemokine production observed in the integrated system. Moreover, we showed that epithelial cells are not passive bystanders during inflammatory gut‐liver crosstalk, but contribute considerably to the overall immune milieu via paracrine interactions with immune cells.

## Discussion

An ability to support multi‐MPS cultures long‐term is desirable for a spectrum of applications in pre‐clinical drug development ranging from disease modeling to PK/PD and toxicity. Interactions between gut and liver under basal and inflammation conditions that we analyze here may be useful in disease modeling, along with capturing physiology related to inflammation‐mediated modulation of drug disposition.

In this study, we demonstrated long‐term (>2 weeks) maintenance of integrated gut and liver functions in baseline conditions. Subtle but significant modulation of cytochrome P450 activities (CYP3A4 and CYP2C9) were observed after 2 weeks of interaction. Transcriptomic analysis of gut and liver in baseline interaction revealed subtle upregulation of cell cycle processes and down‐regulation of pathways involved in hepatic energy metabolism and endogenous/xenobiotic metabolism. Notably, we detected a potential FGF19‐dependent enterohepatic communication as evidenced by inhibition of CYP7A1 expression. Under inflammatory gut‐liver interaction, we detected synergistic amplification of chemokine production, which was in part mediated by TNFα and IFNγ signaling, and showed that intestinal epithelial cells responded to immune cell‐derived signals to influence CXCL9/10/11 and CX3CL1 chemokine production. CXCR3 ligands have been implicated in a number of infectious and chronic diseases. Systemic endotoxemia is commonly observed in IBD patients and tracks with disease activity (Gardiner et al., 1995). In particular, elevated levels of CXCR3 ligands, CXCL9/10/11, have been detected in blood serum as well as intestinal biopsies of IBD patients (Singh et al., 2007). Thus, our gut‐liver MPS platform can identify inter‐organ communication phenomena of both known and hypothesized physiological relevance—motivating follow‐up studies using this controlled environment for mechanistic investigation of the underlying cell‐ and tissue‐level regulation. For instance, we have recently found that these CXCR3 ligands influence the emergence of metastatic breast cancer cells from dormancy in the liver MPS (Clark et al., 2016; manuscript in preparation), demonstrating a novel gut‐liver crosstalk cause of the previously noted effect of inflammatory chemokines in metastatic breast cancer (Kitamura and Pollard, 2015).

While our current MPS does not capture the full spectrum of immunological responses (e.g., adaptive immunity), local immune priming by tissue‐resident cells can shape the potential recruitment, and activation of additional immune cells. The chemokine production observed in the integrated system can target cells of both the innate and adaptive immune system (Fig. 5E), with potential recruitment anticipated based on the chemokines and the corresponding cell receptor profiles. In the current study, we did not specifically track the prospect of immune cell migration in the integrated system. Future investigations may include a more detailed analysis on the trafficking of circulating immune cells for more complex disease modeling.

The liver plays a key role in detoxification, and dysregulated liver function under inflammatory conditions could have systemic implications. Sepsis patients are susceptible to adverse drug reactions due to inflammation‐induced suppression of liver metabolic function, specifically the activity of cytochrome P450 enzyme system (Kim et al., 2011). Our results demonstrated altered mRNA expression of Phase I and Phase II metabolic enzyme in inflammatory gut‐liver crosstalk (Supporting Information Fig. S1 and Table SIII). Thus, accurate prediction of drug PK and PD necessitates the consideration for multi‐organ interaction as well as the physiological context (i.e., health vs. disease). This is especially pertinent for drugs with a narrow therapeutic window because even modest changes to cytochrome P450 activities can precipitate toxicity. An improved understanding of immune‐drug interaction may reduce the incidence of adverse drug reactions in patients with inflammatory diseases.

While multi‐MPS systems hold promise as a new tool for probing human physiology, continued development on hardware design, tissues models as well as computational strategies will be needed to improve in vitro–in vivo translation of experimental observations. For instance, the intestinal epithelial model used here consisted of cell lines, which do not fully capture all the metabolic functions of the primary tissues. We and others are now developing primary intestinal monolayers from patient‐derived enteroid cultures (VanDussen et al., 2015), which, once optimized, can be incorporated to similar multi‐organ platforms for more complex disease modeling and pharmacological studies. The liver module here also lacks stellate cells and liver sinusoidal endothelial cells which contribute to the production and consumption of cytokines, but continued advances in the availability of primary human liver cell preparations will allow these to be incorporated in the future.

Scaling is a significant challenge in the design of MPSs. Broadly, scaling refers two inter‐dependent activities: “on‐platform scaling” and in vitro–in vivo translation (IVIVT) (Stokes et al., 2015). On‐platform scaling, the specification of design and operating parameters to create physiologically representative in vitro systems, is an area of intense investigation. Given that MPS configurations are vastly different from each other and from in vivo organ systems, scaling approaches may need to be tailored for specific applications. Different strategies, such as linear, allometric, and functional scaling, have been proposed to guide MPS design and operations based on human physiology (Abaci and Shuler, 2015; Maass et al., 2017; Maschmeyer et al., 2015b; Ucciferri et al., 2014; Wikswo et al., 2013). On the other hand, IVIVT refers to the process by which experimental results from these minimally essential in vitro systems are converted to in vivo insights.

In the current work, various on‐platform scaling strategies were considered. For intra‐MPS scaling, the design of the individual liver and gut MPS was informed by the major cellular composition of the small intestine and liver tissue in vivo. For inter‐MPS scaling, the surface area of the gut epithelium to hepatocyte number ratio was scaled proportional to the respective in vivo values, giving its importance in determining the absorptive and metabolic function of the gut and liver MPS. Moreover, our flow partitioning scheme was scaled roughly proportional to physiological cardiac output, adjusted for missing tissues.

Another scaling factor commonly emphasized in theoretical analyses of isolated or interconnected MPS systems is the cell‐medium ratio (Abaci and Shuler, 2015; Wikswo et al., 2013). In this work, the volume of medium in the liver compartment is 1.6 mL (6 × 10^5^ hepatocytes and 6 × 10^4^ Kupffer cells), in the gut compartment 0.5 mL apical, and 1.5 mL basal (∼1 × 10^6^ epithelial cells and 1 × 10^5^ dendritic cells), and in the mixer 1 mL. While our system operates with cell‐media ratios that are greater than the blood‐to‐tissue ratio in vivo, this is motivated by the need for both 3D culture and dynamic sampling of the various compartments, such that in vitro PK can be measured directly and translation approaches can be employed to predict in vivo human responses (Tsamandouras et al., 2017). From the perspective of inflammation signaling, our results yielded cytokine/chemokines concentrations on platform that are within the high end of the pathophysiological ranges in patients with inflammatory conditions (see Supplementary Table SXI). This underscores the challenges in scaling MPSs, which are a minimalist representation of organ function, to in vivo physiology. The cytokine concentrations that accrue in the in vitro gut‐liver platform do so in the absence of plasma proteins, proteases, circulating immune cells, and other tissues that can either sequester, degrade, or uptake these molecules. A key point to emphasize is that if cell types particularly relevant to the uptake or production of these molecular factors are missing, such as neutrophils degrading cytokines in the bloodstream (Sarkar and Lauffenburger, 2003), direct linear scaling of media‐volume‐to‐cell ratio based on in vivo blood volume‐to‐tissue ratio would not be appropriate.

Another important difference between in vivo and in vitro is the relationship between convection, diffusion, and reaction of molecular species in vivo compared to MPS. In addition to the role of fluid shear stress, which is generally addressed as an MPS design consideration, individual MPSs may have more (large dimension cell aggregates that rely on diffusion from the fluid channel) or fewer (no endothelial lining) mass transfer barriers compared to tissue, creating functional differences between observed consumption/production rates per cell or gram of tissue in vitro compared to in vivo. For these reasons, so‐called “functional scaling” is emerging as a way to incorporate specific features of MPS operation and behavior relative to in vivo (Maass et al., 2017; Stokes et al., 2015; Wikswo, 2014; Wikswo et al., 2013). The scaling here of the gut and liver MPS incorporates elements of each of these approaches. Given that a complete recapitulation of all tissue functions is not likely feasible in a single in vitro system, continued development of IVIVT strategies is needed to improve the interpretation and extraction of physiological insights by accounting for the missing components.

Overall, our design approach anticipates application of the platform to more complex disease modeling where relatively large complex tissue mass (millions of cells) with high metabolic demands and complex interactions are needed. An example, we have begun pursuing along this avenue is that of addressing resistance to chemotherapeutic drug treatment in breast cancer cells that have metastasized to liver residence (Clark et al., 2016; Edington et al., 2017), and numerous others of importance to drug discovery and development can be envisioned.

This work was supported by the DARPA Microphysiological Systems Program (W911NF‐12‐2‐0039) and NIH UH3TR000696. We are grateful to Emily Geishecker and Catherine Communal for assistance on project management, Rachel Dyer for technical assistance on hepatocyte cultures, Wen‐Han Yu and Anthony Soltis for helpful advice on bioinformatics analysis, and Mohammad R Ebrahimkhani, Timothy Kassis, Nikolaos Tsamandouras, and Christian Alexander Maass for helpful discussion. We thank the MIT BioMicro Center for RNA sequencing.

## Supporting information

Additional supporting information may be found in the online version of this article at the publisher's web‐site.

**Figure S1**. GSEA can reveal more nuanced pathway regulation that might have been masked by strict cut‐offs in gene‐based approach.
**Figure S2**. The cytokine/chemokine production in the inflammatory gut‐liver crosstalk (n=4, mean±SD).
**Figure S3**. TNFα (5 ng/mL) and IFNγ (5 ng/mL) synergistically enhanced CX3CL1 in gut epithelial cells (24 hr).
**Figure S4**. IL‐1β (1 ng/mL) and IFNγ (5 ng/mL) synergistically enhanced CXCL9/10/11 in gut epithelial cells (24 hr).
**Figure S5**. A confocal micrograph of the gut MPS illustrates the polarized epithelium on top of the transwell membrane and the dendritic cells underneath the membrane.
**Figure S6**. A) The liver module contains a rigid, thin (0.25 mm) polystyrene scaffold with 301 microchannels (diameter=0.3 mm) that serve to localize and aggregate primary human hepatocytes and Kupffer cells into miniature liver tissues.
**Figure S7**. Immunofluorescent staining for DNA synthesis marker (EDU) and mucin marker (MUC5AC) revealed an overlap between proliferative population and the MUC5AC‐positive HT29‐MTX cells.
**Table SI**.XXX
**Table SII**.XXX
**Table SIII**. Gene expression changes in liver metabolizing enzymes under inflammatory gut‐liver crosstalk.
**Table SIV**. Common gene sets up‐regulated in gut and liver during interaction.
**Table SV**. Common gene sets down‐regulated in gut and liver during interaction.
**Table SVI**. Unique gene sets up‐regulated in liver during inflammatory gut‐liver crosstalk.
**Table SVII**. Unique gene sets up‐regulated in gut during inflammatory gut‐liver crosstalk.
**Table SVIII**. Unique gene sets down‐regulated in liver during inflammatory gut‐liver crosstalk.
**Table SIX**. Unique gene sets down‐regulated in gut during inflammatory gut‐liver crosstalk.
**Table SX**. TLR expression (Log10 expression normalized to GAPDH).
**Table SXI**. Comparison of cytokine/chemokine concentrations obtained on the gut‐liver interaction platform versus the in vivo values in patients with systemic inflammation.
