# Supplementary material for: Integrated gut/liver microphysiological systems elucidates inflammatory inter‐tissue crosstalk
Source: Biotechnol Bioeng. 2017 Jul 27;114(11):2648–59. doi: 10.1002/bit.26370 (PMC5614865; doi:10.1002/bit.26370)
Supplement: Supplementary file 1 — Figure S1. GSEA can reveal more nuanced pathway regulation that might have been masked by strict cut‐offs in gene‐based approach. Figure S2. The cytokine/chemokine production in the inflammatory gut‐liver crosstalk (n=4, mean±SD). Figure S3. TNFα (5 ng/mL) and IFNγ (5 ng/mL) synergistically enhanced CX3CL1 in gut epithelial cells (24 hr). Figure S4. IL‐1β (1 ng/mL) and IFNγ (5 ng/mL) synergistically enhanced CXCL9/10/11 in gut epithelial cells (24 hr). Figure S5. A confocal micrograph of the gut MPS illustrates the polarized epithelium on top of the transwell membrane and the dendritic cells underneath the membrane. Figure S6. A) The liver module contains a rigid, thin (0.25 mm) polystyrene scaffold with 301 microchannels (diameter=0.3 mm) that serve to localize and aggregate primary human hepatocytes and Kupffer cells into miniature liver tissues. Figure S7. Immunofluorescent staining for DNA synthesis marker (EDU) and mucin marker (MUC5AC) revealed an overlap between proliferative population and the MUC5AC‐positive HT29‐MTX cells. Table SI.XXX Table SII.XXX Table SIII. Gene expression changes in liver metabolizing enzymes under inflammatory gut‐liver crosstalk. Table SIV. Common gene sets up‐regulated in gut and liver during interaction. Table SV. Common gene sets down‐regulated in gut and liver during interaction. Table SVI. Unique gene sets up‐regulated in liver during inflammatory gut‐liver crosstalk. Table SVII. Unique gene sets up‐regulated in gut during inflammatory gut‐liver crosstalk. Table SVIII. Unique gene sets down‐regulated in liver during inflammatory gut‐liver crosstalk. Table SIX. Unique gene sets down‐regulated in gut during inflammatory gut‐liver crosstalk. Table SX. TLR expression (Log10 expression normalized to GAPDH). Table SXI. Comparison of cytokine/chemokine concentrations obtained on the gut‐liver interaction platform versus the in vivo values in patients with systemic inflammation. [file BIT-114-2648-s001.docx]

**Materials and Methods:**

All hardware components, with the exception of the polyurethane membranes, are reusable.

*Cleaning*

Before each use, the polysulfone top plate was cleaned and sterilized.  First, the plate was submerged in 10% bleach for 30-60 minutes, followed by a short rinse in distilled water.  To remove any residual contaminants, the top plate was sonicated in 10% solution 7x solution (MP Biomedicals #MP0976680HP) for 15 minutes.  Two subsequent 15-minute sonication cycles in fresh DI water ensured removal of all surfactant before a final DI water rinse.  The plate was then air dried, sealed in a sterilization bag, and autoclaved.

The pneumatic plate does not require formal sterilization, but prior to assembly it was wiped thoroughly with a kimwipe sprayed with 70% ethanol, taking care to remove any dust or particles from the sealing areas that contact the membrane.

Polyurethane (PU) membranes (American Polyfilm Inc), 50 microns thick, were stretched on tension rings to maintain a constant tension.  They were laser cut with the symmetric pattern of screw holes on the pneumatic plate.  After laser cutting, they were rinsed in 10% 7x solution and then with excess DI water.  After air drying, they were sterilized with ethylene oxide gas and allowed to degas in a chemical fume hood for 24 hours.

*Assembly*

The fluidic plate, pneumatic plate, and membrane were assembled in a biosafety cabinet.  Before assembly, a sterile microplate lid was taped onto the fluidic plate to protect the sterility of the cell culture region.  The layers can then be assembled upside down to aid with visual alignment through the acrylic plate.  Once the alignment pins mate with the fluidic plate, the platform can be carefully removed from the hood, keeping pressure to maintain the seal between the plates.  Screws can be inserted and tightened in a nonsterile environment as long as the plates are not separated.  Two fully assembled platforms were daisy-chained with short tubing connecting straight across to the corresponding ports. Daisy-chained platforms can be easily transported with a large metal tray (15 by 10 in.).

Platforms were assembled at day (-4) prior to the start of the interaction experiment. Sterile platforms were primed with 1% bovine serum albumin (BSA, Sigma A9576) and 1x Penicillin-Streptomycin (P/S, Gibco™ 15140-148) in PBS. Pump function and tubing connections were visually confirmed by pumping from the mixer to each dry compartment, then by running the recirculation pumps backwards to clear all air from the channels.  Spillways were manually wetted with small volumes to ensure spillway operation.  Platforms were run overnight in the incubator to passivate and confirm full operation before the addition of cells.

**Platform operation**

Supplemental table 1 and 2 tabulated the compartmental volume and flow rates used based on the flow circuit specified in Fig 1B. The systemic flow rate (output from the mixer) was computationally determined to be 15 mL/day to enable proper mixing of endogenously produced biomolecule. The computational methodology was adapted from (Yu et al. 2015). The flow partitioning from the mixer to gut and liver MPS was scaled proportional to physiological cardiac output to gut and liver in human (Brown et al. 1997). Specifically, 25% and 75% of the systemic flow was directed to the liver and gut compartment, respectively. The effluent from the gut fed to the liver, representing portal circulation. Values for the operational flow rates and nominal volumes are tabulated in Table S1 and Table S2.

| Table S1: | | |
| --- | --- | --- |
| **Compartments** | | **Flow rates** (µL/s) |
| Mixer | self-circ | 1.0 |
|  | mixer-gut | 0.13 |
|  | mixer-liver | 0.043 |
| Liver | self-circ | 1.0 |
| Gut | self-circ, basal | 0.25 |

| Table S2: | | |
| --- | --- | --- |
| **Compartments** | | **Volume**  (mL) |
| Mixer | | 1.0 |
| Liver | | 1.6 |
| Gut | Apical | 0.5 |
|  | Basal | 1.5 |

**Albumin assay**

To evaluate the health of the liver, samples from all compartments were taken at every media change (every 72 hours) and assayed for albumin via ELISA (Bethyl Laboratories, E80-129).

**Cytochrome P450 assay**

Various Cytochrome P450 (CYP) enzyme activities were measured using a developed CYP cocktail assay (Pillai et al. 2013). Briefly, a cocktail of CYP substrates was added to liver compartment for a one-hour incubation, and the supernatant was collected for downstream processing. Substrate-specific metabolite production was analyzed using mass spec.

**Barrier function/transepithelial electrical resistance (TEER)**

TEER measurement was performed using the EndOhm-12 chamber with an EVOM2 meter (World Precision Instruments). The samples and the EndOhm chamber were kept warm at ~37°C on a hot plate. Temperature was rigorously maintained during TEER measurement to minimize variability.

**Mucin quantification**

Secreted mucin in the apical gut compartment was measured using an Alcian Blue assay. The mucin quantification protocol was adapted from (Hall et al. 1980). Briefly, media from apical was collected in low-binding tubes, and spun down at 10,000 g for 5 minutes, and the supernatant was collected and stored at -80 °C for subsequent analysis. Mucin secretion was quantified against a standard of mucin (Sigma M3895) dissolved in culture medium. Samples and standards were incubated in a 96-well plate in a 3:1 mix of sample to Alcian Blue solution (Richard Allen Scientific) for two hours. After incubation, plates were centrifuged at 1640g for 30 minutes at room temperature. Supernatant was removed by inverting the plates onto dry paper towels. Samples were rinsed twice with wash buffer (40% (v/v) of ethanol and 60% (v/v) of 0.1M sodium acetate buffer containing 25mM MgCl­_2_ at pH 5.8), with a 10-minute centrifugation step after each rinse. After second spin, supernatant was removed and samples were dissolved with 10% SDS in distilled water. Plates typically required shaking or pipetting to fully resuspend samples. If bubbles formed during resuspension, plates were centrifuged again for about 5 minutes prior to absorbance measurement on a Spectramax m3/m2e at 620nm.

**FGF19 quantification**

FGF19 production was quantified using a ELISA (BioVendor).

**Multiplex cytokine/chemokine assays**

Cytokine levels were measured using multiplex cytokine assays, 37-plex human inflammation and 40-plex panel chemokine panels (Bio-Rad Laboratories, Inc., Hercules, CA, USA). Briefly, media samples were collected in low-binding tubes, spun down at 10,000 g for 5 mins to remove cell debris, and the supernatant was stored in -80 °C. Samples were measured at multiple dilutions to ensure the measurements were within the linear dynamic range of the assay. To minimize non-specific binding to beads, BSA was added to achieve a final concentration of 5 mg/mL in all samples. We reconstituted the protein standard in the same media and serially diluted the protein stock to generate an 8-point standard curve. Assays were run on a Bio-Plex 3D Suspension Array System (Bio-Rad Laboratories, Inc.). Data were collected using the xPONENT for FLEXMAP 3D software, version 4.2 (Luminex Corporation, Austin, TX, USA). The concentration of each analyte was determined from a standard curve, which was generated by fitting a 5-parameter logistic regression of mean fluorescence on known concentrations of each analyte (Bio-Plex Manager software).

To obtained the total production amount per platform, the concentration values were normalized by compartmental volume and added up across all compartments (mixer, gut, liver) in each platform.

**Multivariate analysis**

Hierarchical clustering and principal component analysis was perform using Matlab R2016a software (Mathworks, Natick MA). Cytokine data were normalized by mean-centering and variance scaling prior to clustering and principal component analysis.

**RNA extraction, cDNA library preparation and next generation sequencing**

For both the baseline and inflamed conditions (at Day 3, n=4), intestinal and hepatic tissues was taken out of the platforms, and mRNA was extracted using the PureLink RNA mini kit (ThermoFisher, 12183018A). Total RNA was analyzed and quantified using the Fragment Analyzer (Advanced Analytical), and cDNA was generated using the SMART-Seq v3 kit (Clontech). After cDNA fragmentation (Covaris S2), cDNA was end-repaired and adaptor-ligated using the SPRI-works Fragment Library System I (Beckman Coulter Genomics). Adaptor-ligated cDNA was then indexed during PCR amplification, and the resulting libraries were quantified using the Fragment Analyzer and qPCR before being sequenced on the Illumina HiSeq 2000. 40-50 nt single-end read with an average depth of 15-20 million or 5 million reads per sample were sequenced for the baseline and inflamed conditions respectively.

**RNAseq data analysis**

The FASTQ files were generated from the sequencing runs. The resultant reads were aligned to the human reference genome (GRch37/hg19) using Tophat (version 2.0.12) (Kim et al. 2013) to identify reads that map to known transcripts, accounting for splice junctions. HTSeq was used to determine the number of read counts uniquely overlap with known genomic features (Anders et al. 2015).

**Differential gene expression analysis**

To identify significantly altered genes in isolation vs interaction conditions, differential gene analysis of count data was performed using DESeq2 (Version 1.12.3) in R (Love et al. 2014). Only genes with greater than 1 cpm (count per million) in at least 4 replicates, were included in the analysis. Multiple testing correction was performed using the procedure of Benjamini and Hochberg. Genes with an adjusted P-value below a FDR cutoff of 0.05 were considered significant.

**GO analysis**

GOSeq R packages (Young et al. 2010) was used to determine the over-represented biological of the differentially expressed genes (FDR-adjusted P-values<0.05).

**Gene set enrichment analysis and visualization**

GSEA (version 2.2.3) was performed to identify differentially regulated gene sets in isolation versus interaction, as describe in (Subramanian et al. 2005). To stabilize variance, the normalized count data were processed using a regularized logarithm transformation in DESeq2 (Love et al. 2014). The signal-to-noise metric was used to generate the ranked list of genes. Canonical pathway gene sets from Molecular Signatures Database (c2.cp.v5.2) were used, which is a collection of curated genes sets from multiple databases (e.g., Reactome, KEGG, BioCarta, PID). The empirical P-values for each enrichment score were calculated relative to the null distribution of enrichment scores, which was computed via 1000 gene set permutations. Gene sets with nominal P-value <0.01 and q-value <0.05 were considered significant. Enrichment map (Merico et al. 2010), a Cytoscape plugin, was used to visualize the overlaps between significant gene sets and to facilitate the systematic interpretation of the interdependencies among different biological processes.

**
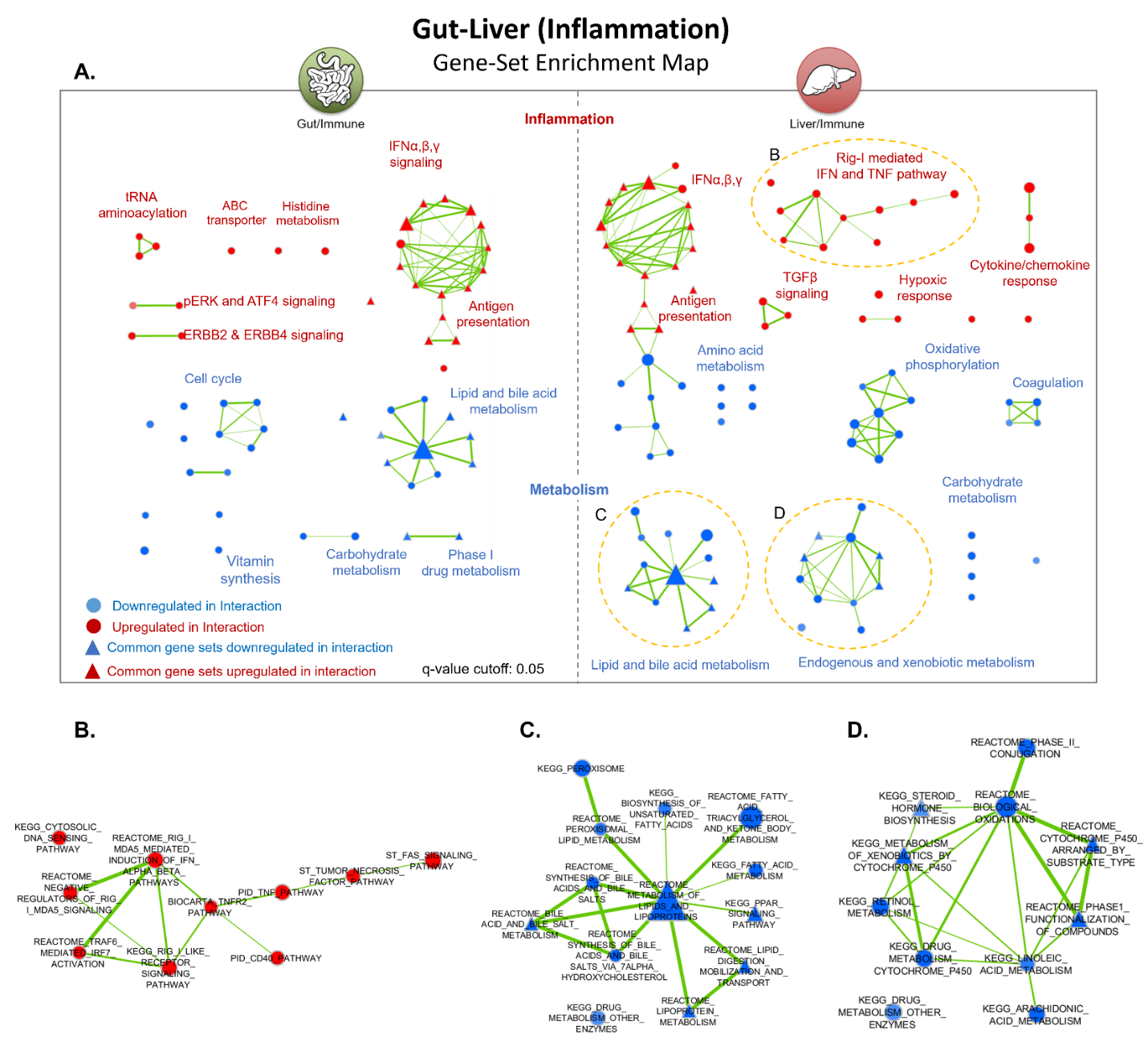
**

**Fig S1:** GSEA can reveal more nuanced pathway regulation that might have been masked by strict cut-offs in gene-based approach. Generally, GSEA results were largely consistent with GO analysis outcomes, but with greater interpretability and generality. We obtained consensus clusters of gene sets from different databases, which contained overlapping but distinct groups of genes that define major biological processes. Specifically, inflammation-related pathways centered around IFNα/β/γ signaling were up-regulated and metabolic processes involving cholesterol and lipid metabolism were down-regulated in both the gut and liver in interaction (The gene sets are tabulated in Supplementary Table S4-S9). A) Gene set enrichment map illustrates the significantly modulated gene sets during inflammatory gut-liver crosstalk. Biological pathway labels were assigned to functionally-related gene sets manually. The triangular nodes correspond to gene sets that were concurrently altered in both the gut and liver tissues during interaction. The circular nodes indicate the unique gene sets differentially changed in the gut and liver tissues during interaction. Red and blue differentiates the up- and down-regulated gene sets respectively. The edges represent the degree of gene overlap of different gene sets. B) Gene sets involved in RIG-I mediated IFN and TNF signaling. C) Gene sets involved in cholesterol and lipid metabolism. D) Gene sets involved in drug metabolism.


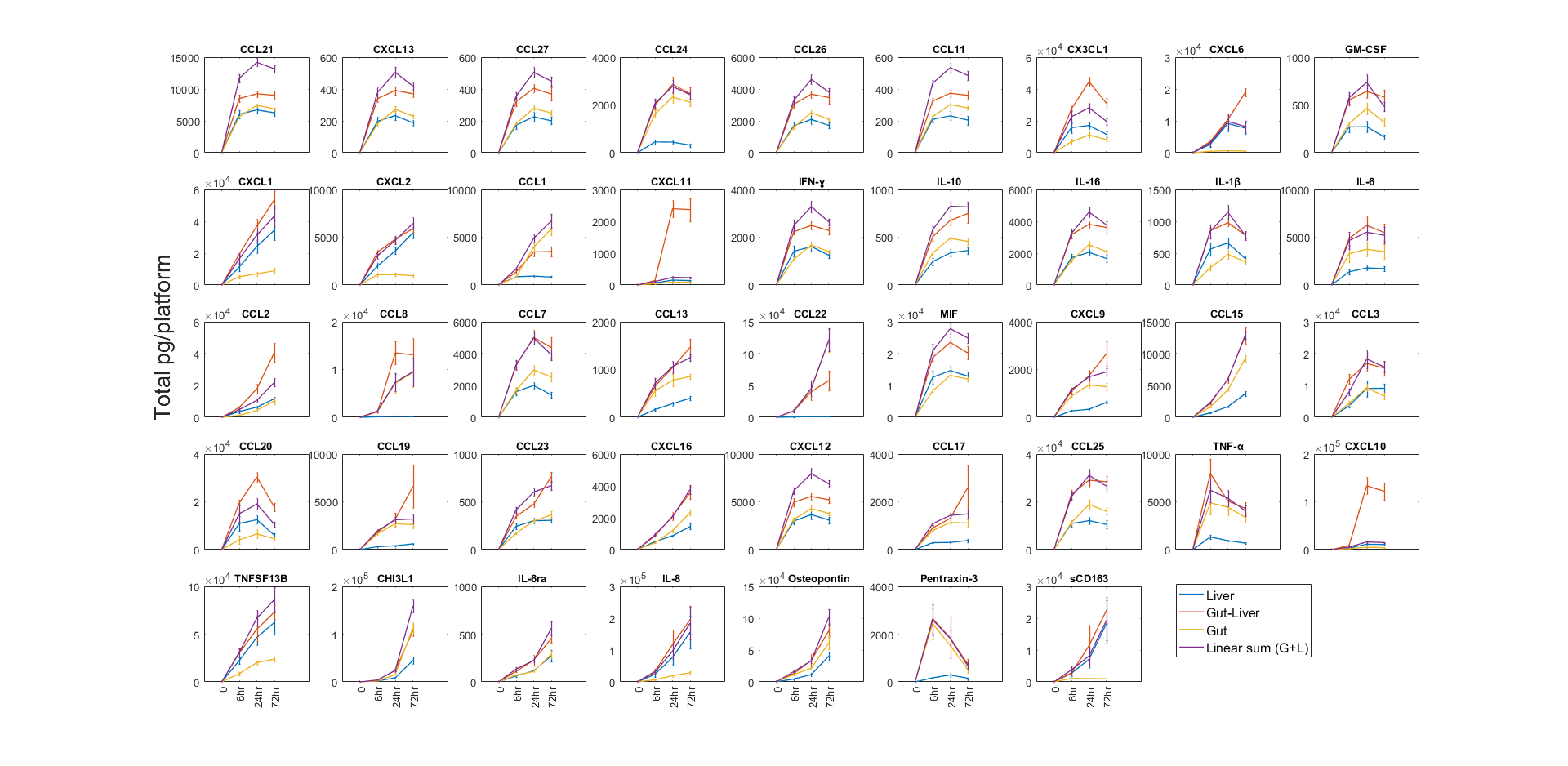


**Fig S2:** The cytokine/chemokine production in the inflammatory gut-liver crosstalk (n=4, mean±SD).





**Fig S3:** TNFα (5 ng/mL) and IFNγ (5 ng/mL) synergistically enhanced CX3CL1 in gut epithelial cells (24 hr). The dash line in each plot indicates the theoretical chemokine level from summing the TNFα- and IFNγ-only conditions (n=3). mean±SD


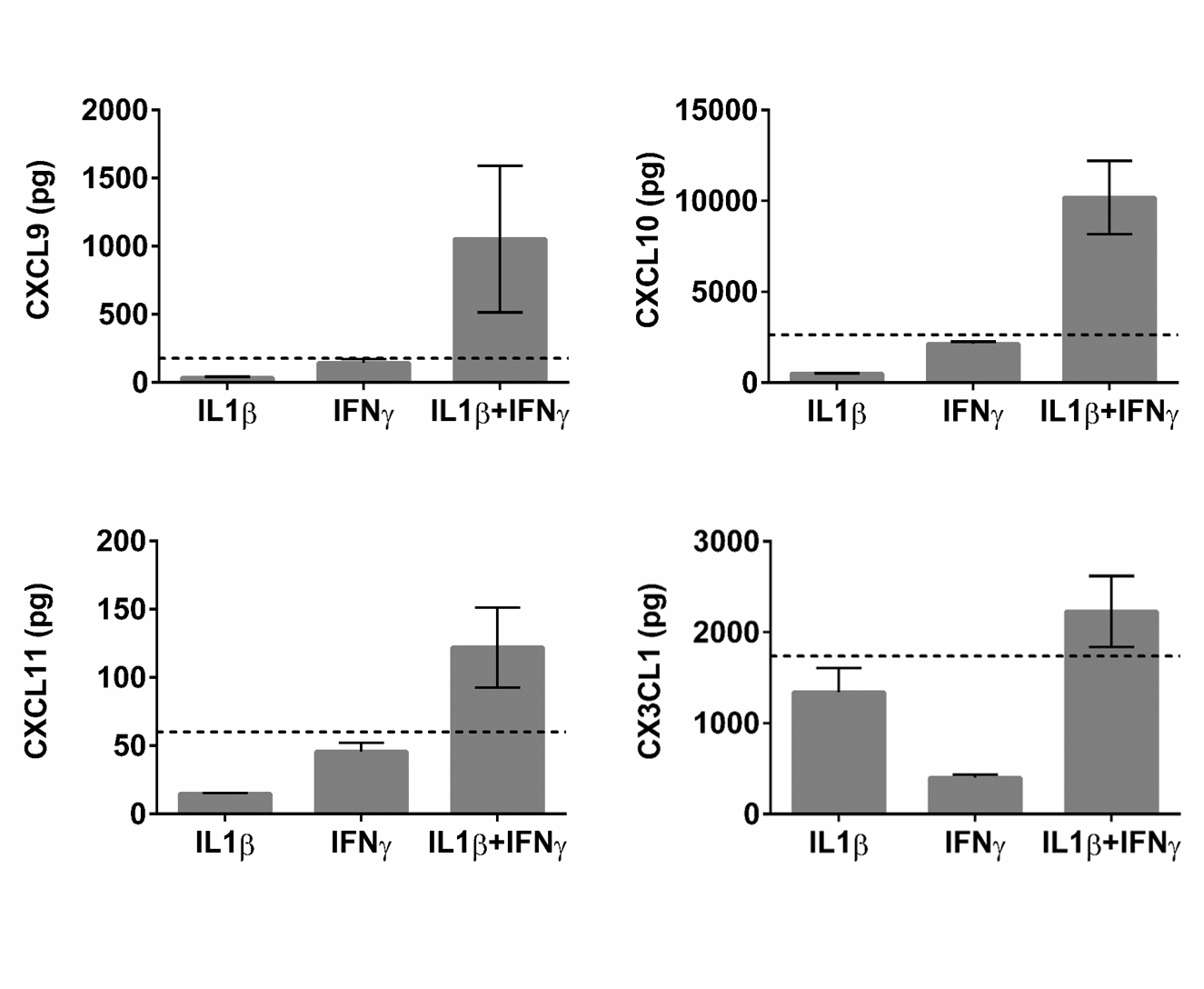


**Fig S4:** IL-1β (1 ng/mL) and IFNγ (5 ng/mL) synergistically enhanced CXCL9/10/11 in gut epithelial cells (24 hr). The dash line in each plot indicates the theoretical chemokine level from summing the IL-1β- and IFNγ-only conditions (n=3). mean±SD.


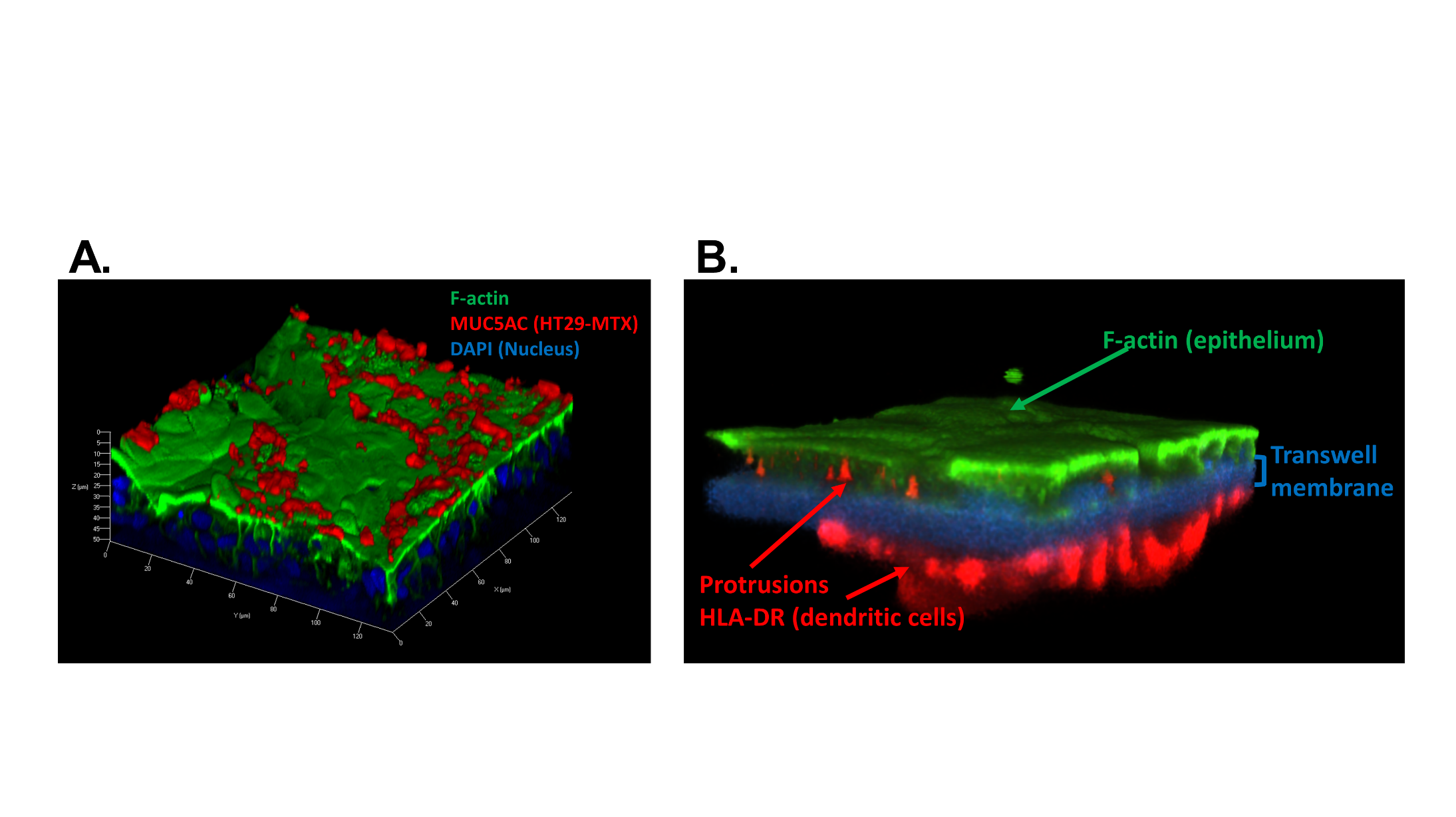


**Fig S5:** A confocal micrograph of the gut MPS illustrates the polarized epithelium on top of the transwell membrane and the dendritic cells underneath the membrane. A) The epithelium is composed of a co-culture of Caco2 and mucus-producing, MUC5AC-positive, HT29-MTX cells. B) Dendritic cells are located underneath the Transwell membrane, and in focal regions, they extend processes through the membrane to intercalate with the epithelial layer above.


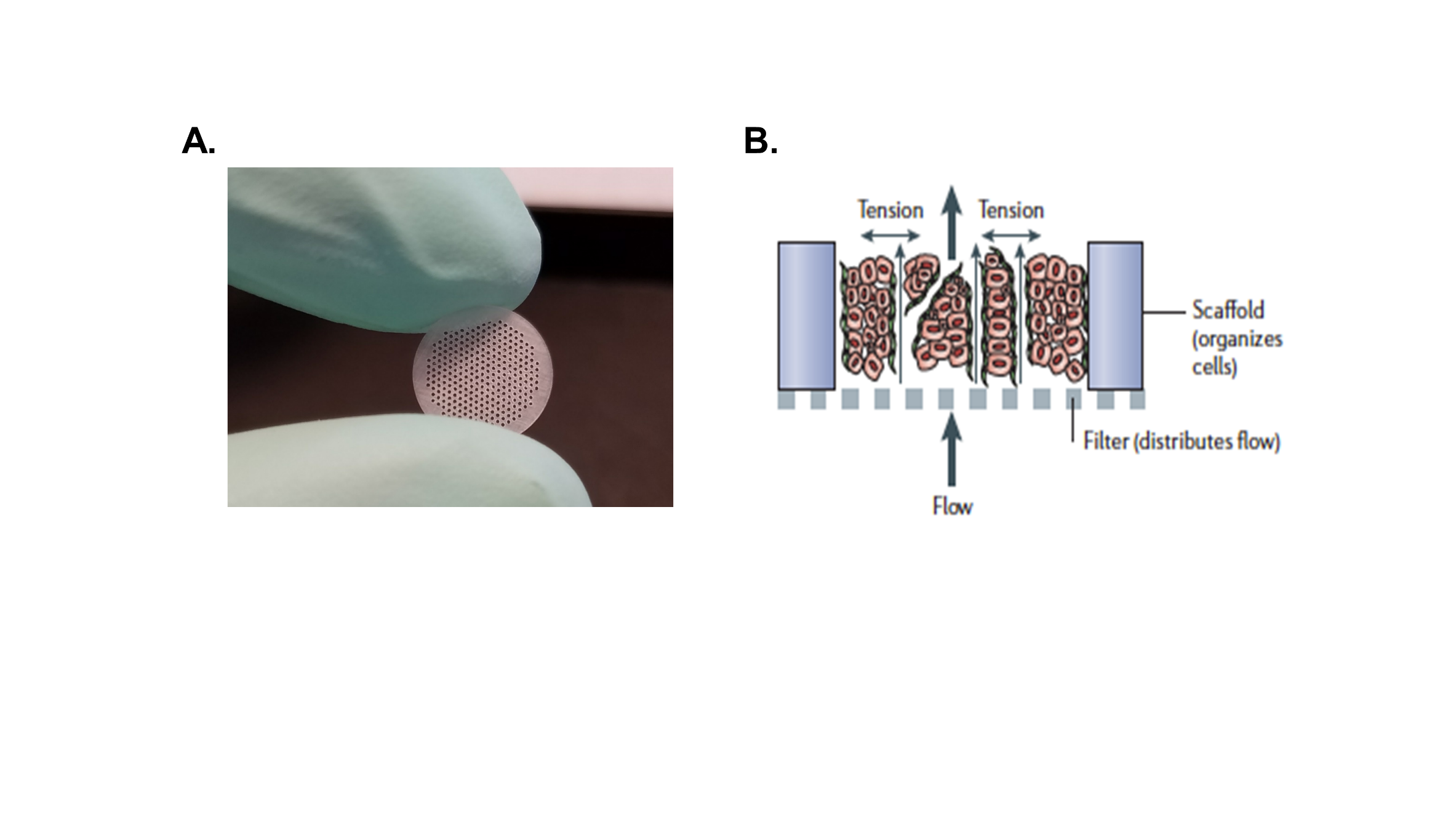


**Fig S6:** A) The liver module contains a rigid, thin (0.25 mm) polystyrene scaffold with 301 microchannels (diameter=0.3 mm) that serve to localize and aggregate primary human hepatocytes and Kupffer cells into miniature liver tissues. B) A schematic illustrates the organization of liver tissue in a single microchannel on the liver scaffold under perfusions.


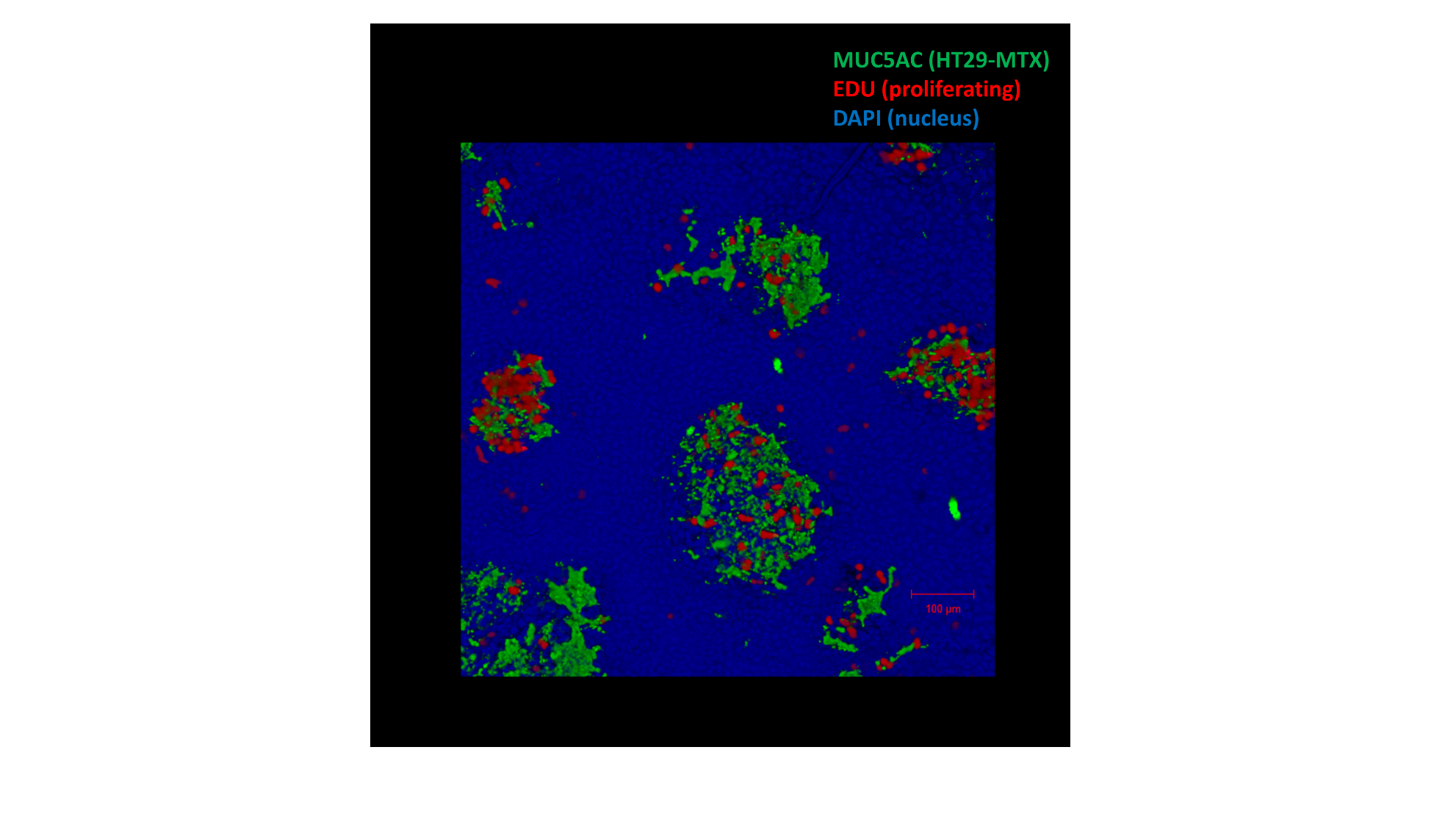


**Fig S7:** Immunofluorescent staining for DNA synthesis marker (EDU) and mucin marker (MUC5AC) revealed an overlap between proliferative population and the MUC5AC-positive HT29-MTX cells.

| **Table S3:** Gene expression changes in liver metabolizing enzymes under inflammatory gut-liver crosstalk | | | |
| --- | --- | --- | --- |
| **Genes** | **log2FoldChange (Interaction/Isolation)** | **P** | **Adj. P** |
| CYP1A2 | -1.34 | 3.9E-13 | 1.1E-11 |
| CYP2C9 | -0.76 | 3.1E-20 | 1.8E-18 |
| CYP2C19 | -1.12 | 4.2E-07 | 6.0E-06 |
| CYP2D6 | -1.07 | 1.3E-28 | 1.4E-26 |
| CYP3A4 | -1.98 | 1.7E-70 | 1.0E-67 |
| CYP3A5 | -0.49 | 1.2E-09 | 2.4E-08 |

| **Table S4:** Common gene sets up-regulated in gut and liver during interaction | |  | | |  |
| --- | --- | --- | --- | --- | --- |
| **Pathways** | | | **Liver: q-val** | **Gut : q-val** | |
| IFN  signaling | Reactome_Interferon_alpha_beta_signaling | | 0.0E+00 | 0.0E+00 | |
|  | Reactome_Interferon _gamma_signaling | | 0.0E+00 | 0.0E+00 | |
|  | Reactome_Interferon _signaling | | 0.0E+00 | 0.0E+00 | |
| Cytokine signaling | Reactome_cytokine_signaling_in_immune_system | | 0.0E+00 | 3.0E-03 | |
| Antigen processing | Kegg_antigen_processing_and_presentation | | 1.0E-03 | 1.0E-03 | |
|  | Reactome_antigen_presentation_folding_assembly_and_peptide_loading_of_class_I_MHC | | 2.0E-03 | 3.0E-03 | |
|  | Reactome_antigen_processing_cross_presentation | | 6.0E-03 | 2.5E-02 | |
|  | Reactome_ER_phagosome_pathway | | 7.0E-03 | 4.0E-03 | |
| Immune processes | Kegg_intestinal_immune_network_for_IGA_production | | 1.8E-02 | 2.5E-02 | |
|  | Reactome_immunoregulatory_interactions_between_a_lymphoid_and_a_non_lymphoid_cell | | 4.0E-03 | 2.4E-02 | |
|  | Kegg_allograft_rejection | | 1.0E-03 | 0.0E+00 | |
|  | Kegg_autoimmune_thyroid_disease | | 2.0E-03 | 0.0E+00 | |
|  | Kegg_viral_myocarditis | | 2.0E-03 | 4.0E-03 | |
|  | Kegg_graft_versus_host_disease | | 3.0E-03 | 0.0E+00 | |
|  | Kegg_Type_I_diabetes_mellitus | | 7.0E-03 | 0.0E+00 | |

| **Table S5:** Common gene sets down-regulated in gut and liver during interaction | | |  | |  |
| --- | --- | --- | --- | --- | --- |
| **Pathways** | | **Liver: q-val** | | **Gut : q-val** | |
| Endogeneous and xenobiotic metabolism | Reactome_cytochrome_p450_arranged_by_substrate_type | 0.0E+00 | | 3.3E-02 | |
|  | Reactome_phase I_functionalization_of_compounds | 0.0E+00 | | 4.4E-02 | |
|  | Kegg_metabolism_of_xenobiotics_by_cytochrome_p450 | 0.0E+00 | | 4.6E-02 | |
| Lipid metabolism | Kegg_PPAR_signaling_pathway | 0.0E+00 | | 4.0E-03 | |
|  | Reactome_lipid_digestion_mobilization_and_transport | 4.0E-03 | | 3.9E-02 | |
|  | Reactome_lipoprotein_metabolism | 1.0E-02 | | 4.3E-02 | |
|  | Reactome_metabolism_of_lipids_and_lipoproteins | 1.0E-03 | | 8.0E-03 | |
| Steroid and bile acid metabolism | Kegg_steroid_hormone_biosynthesis | 1.1E-02 | | 0.0E+00 | |
|  | Reactome_bile_acid_and_bile_salt_metabolism | 0.0E+00 | | 3.2E-02 | |

| **Table S6:** Unique gene sets up-regulated in liver during inflammatory gut-liver crosstalk | | |
| --- | --- | --- |
| **Gene sets** | **Liver: q-val** | |
| Biocarta_TNFR2_pathway |  | 8.0e-03 |
| St_tumor_necrosis_factor_pathway |  | 3.0e-03 |
| PID_TNF_pathway | 5.0E-03 | |
| Reactome_chemokine_receptors_bind_chemokines |  | 8.0e-03 |
| Kegg_cytokine_cytokine_receptor_interaction |  | 0.0e+00 |
| Kegg_rig_i_like_receptor_signaling_pathway |  | 3.1e-02 |
| Kegg_cytosolic_dna_sensing_pathway |  | 8.0e-03 |
| Reactome_negative_regulators_of_rig_i_MDA5_signaling |  | 3.1e-02 |
| Naba_secreted_factors |  | 1.6e-02 |
| PID_CD40_pathway |  | 2.0e-03 |
| PID_hif1_tfpathway |  | 3.0e-03 |
| PID_hif2pathway |  | 2.0e-03 |
| PID_il23_pathway |  | 3.6e-02 |
| Kegg_primary_immunodeficiency |  | 1.7e-02 |
| Reactome_antiviral_mechanism_by_ifn_stimulated_genes |  | 1.0e-03 |
| Reactome_o_linked_glycosylation_of_mucins |  | 2.0e-03 |
| Reactome_regulation_of_hypoxia_inducible_factor_hif_by_oxygen |  | 4.0e-03 |
| Reactome_rig_i_mda5_mediated_induction_of_ifn_alpha_beta_pathways |  | 1.0e-02 |
| Reactome_signaling_by_tgf_beta_receptor_complex |  | 2.4e-02 |
| Reactome_smad2_smad3_smad4_heterotrimer_regulates_transcription |  | 6.0e-03 |
| Reactome_traf6_mediated_irf7_activation |  | 1.1e-02 |
| Reactome_transcriptional_activity_of_smad2_smad3_smad4_heterotrimer |  | 8.0e-03 |
| St_fas_signaling_pathway |  | 1.6e-02 |

| **Table S7:** Unique gene sets up-regulated in gut during inflammatory gut-liver crosstalk | | |  |
| --- | --- | --- | --- |
| **Gene sets** | **Gut: q-val** | |  |
| PID_IL12_2pathway |  | 2.4E-02 |  |
| Kegg_abc_transporters |  | 1.4E-02 |  |
| Reactome_amino_acid_synthesis_and_interconversion_transamination |  | 2.5E-02 |  |
| Kegg_aminoacyl_trna_biosynthesis |  | 0.0E+00 |  |
| Reactome_cytosolic_trna_aminoacylation |  | 0.0E+00 |  |
| Reactome_trna_aminoacylation |  | 0.0E+00 |  |
| Kegg_cell_adhesion_molecules_cams |  | 2.6E-02 |  |
| Kegg_histidine_metabolism |  | 2.1E-02 |  |
| Reactome_activation_of_genes_by_atf4 |  | 2.0E-02 |  |
| Reactome_perk_regulated_gene_expression |  | 2.6E-02 |  |
| Reactome_PI3K_events_in_erbb2_signaling |  | 4.0E-03 |  |
| Reactome_PI3K_events_in_erbb4_signaling |  | 2.4E-02 |  |

| **Table S8:** Unique gene sets down-regulated in liver during inflammatory gut-liver crosstalk | | | |
| --- | --- | --- | --- |
| **Gene sets** | **Liver: q-val** | | |
| Biocarta_ami_pathway | | | 3.8E-02 |
| Biocarta_intrinsic_pathway | | | 3.0E-03 |
| Kegg_alanine_aspartate_and_glutamate_metabolism | | | 4.2E-02 |
| Kegg_arachidonic_acid_metabolism | | | 8.0E-03 |
| Kegg_arginine_and_proline_metabolism | | | 3.0E-03 |
| Kegg_beta_alanine_metabolism | | | 2.0E-03 |
| Kegg_biosynthesis_of_unsaturated_fatty_acids | | | 1.4E-02 |
| Kegg_butanoate_metabolism | | | 0.0E+00 |
| Kegg_citrate_cycle_tca_cycle | | | 0.0E+00 |
| Kegg_complement_and_coagulation_cascades | | | 8.0E-03 |
| Kegg_drug_metabolism_cytochrome_p450 | | | 0.0E+00 |
| Kegg_drug_metabolism_other_enzymes | | | 9.0E-03 |
| Kegg_fatty_acid_metabolism | | | 0.0E+00 |
| Kegg_glycine_serine_and_threonine_metabolism | | | 0.0E+00 |
| Kegg_glycolysis_gluconeogenesis | | | 0.0E+00 |
| Kegg_glyoxylate_and_dicarboxylate_metabolism | | | 1.3E-02 |
| Kegg_histidine_metabolism | | | 1.4E-02 |
| Kegg_linoleic_acid_metabolism | | | 5.0E-03 |
| Kegg_lysine_degradation | | | 3.0E-03 |
| Kegg_oxidative_phosphorylation | | | 0.0E+00 |
| Kegg_parkinsons_disease | | | 1.0E-03 |
| Kegg_peroxisome | | | 0.0E+00 |
| Kegg_propanoate_metabolism | | | 0.0E+00 |
| Kegg_proximal_tubule_bicarbonate_reclamation | | | 2.3E-02 |
| Kegg_pyruvate_metabolism | | | 0.0E+00 |
| Kegg_retinol_metabolism | | | 0.0E+00 |
| Kegg_tryptophan_metabolism | | | 2.0E-03 |
| Kegg_tyrosine_metabolism | | | 0.0E+00 |
| Kegg_valine_leucine_and_isoleucine_degradation | | | 0.0E+00 |
| PID_hnf3b_pathway | | | 1.0E-03 |
| Reactome_biological_oxidations | | | 0.0E+00 |
| Reactome_branched_chain_amino_acid_catabolism | | | 0.0E+00 |
| Reactome_citric_acid_cycle_tca_cycle | | | 4.0E-03 |
| Reactome_fatty_acid_triacylglycerol_and_ketone_body_metabolism | | | 2.0E-03 |
| Reactome_formation_of_fibrin_clot_clotting_cascade | | | 3.0E-03 |
| Reactome_metabolism_of_amino_acids_and_derivatives | | | 0.0E+00 |
| Reactome_peroxisomal_lipid_metabolism | | | 3.1E-02 |
| Reactome_phase_ii_conjugation | | | 6.0E-03 |
| Reactome_pyruvate_metabolism_and_citric_acid_tca_cycle | | | 2.0E-03 |
| Reactome_respiratory_electron_transport | | | 0.0E+00 |
| Reactome_respiratory_electron_transport_atp_synthesis_by_chemiosmotic_coupling_and_heat_production_by_uncoupling_proteins_ | | 0.0E+00 | |
| Reactome_synthesis_of_bile_acids_and_bile_salts | | 0.0E+00 | |
| Reactome_synthesis_of_bile_acids_and_bile_salts_via_7alpha_hydroxycholesterol | | 0.0E+00 | |
| Reactome_tca_cycle_and_respiratory_electron_transport | | 0.0E+00 | |

| **Table S9:** Unique gene sets down-regulated in gut during inflammatory gut-liver crosstalk | |
| --- | --- |
| **Gene sets** | **Gut : q-val** |
| Biocarta_TNFR2_pathway | 4.8E-02 |
| Kegg_DNA_replication | 3.6E-02 |
| Kegg_pantothenate_and_coa_biosynthesis | 3.2E-02 |
| Kegg_pentose_and_glucuronate_interconversions | 3.8E-02 |
| Kegg_steroid_biosynthesis | 0.0E+00 |
| Kegg_terpenoid_backbone_biosynthesis | 8.0E-03 |
| PID_aurora_b_pathway | 3.6E-02 |
| PID_hif1_tfpathway | 4.0E-02 |
| Reactome_activation_of_atr_in_response_to_replication_stress | 4.2E-02 |
| Reactome_activation_of_the_pre_replicative_complex | 3.2E-02 |
| Reactome_cholesterol_biosynthesis | 0.0E+00 |
| Reactome_deposition_of_new_cenpa_containing_nucleosomes_at_the_centromere | 9.0E-03 |
| Reactome_DNA_strand_elongation | 7.0E-03 |
| Reactome_e2f_mediated_regulation_of_dna_replication | 3.3E-02 |
| Reactome_fatty_acyl_coa_biosynthesis | 4.0E-03 |
| Reactome_formation_of_tubulin_folding_intermediates_by_cct_tric | 4.8E-02 |
| Reactome_g1_s_specific_transcription | 2.9E-02 |
| Reactome_g2_m_checkpoints | 2.9E-02 |
| Reactome_transport_of_vitamins_nucleosides_and_related_molecules | 6.0E-03 |
| Reactome_triglyceride_biosynthesis | 5.0E-03 |

| **Table S10: TLR expression (Log10 expression normalized to GAPDH)** | | | | |  |
| --- | --- | --- | --- | --- | --- |
| **Cell types** | **TLR1** | **TLR2** | **TLR3** | **TLR4** | **TLR5** |
| Primary human hepatocytes (thawed) | 179.6 | 48.0 | 332.0 | 12.0 | 13.7 |
| Primary human hepatocyte after 4 days in culture | 299.2 | 104.2 | 314.4 | 50.4 | 13.2 |
| Primary Kupffer cells (thawed) | 3496.4 | 10713.5 | 83.7 | 2753.7 | 24.5 |

| **Table S11: Comparison of cytokine/chemokine concentrations obtained on the gut-liver interaction platform versus the *in vivo* values in patients with systemic inflammation** | | | | | | | |
| --- | --- | --- | --- | --- | --- | --- | --- |
|  | ***On Platform***  (mixer, pg/mL) | | ***In Vivo***  (serum or plasma, pg/mL) | | | | |
|  | Baseline | Inflamed | | Healthy | Inflammatory bowel disease | Experimental  endotoxemia | Clinical systemic infection |
| **TNFα** | 66 | 1269 | | 3 (0.9-27)^b^;  30 (24-36)^e^;  0 (0-1.9)^k^ | 50-450^c^;  39 (23-56)^e^ | 6-16^f^;  68-1374^g^;  680-1040^i^ | 2 (0-3)^k^ |
| **IL-6** | 41 | 1588 | | 3 (0.2-38)^b^;  12 (10-15)^e^;  10 (5-20)^k^ | 100-5000^d^;  16 (11-31)^e^ | 10-70^f^;  72-2820^g^;  900 – 1400^i^ | 158 (32-4006)^k^ |
| **IFNγ** | 234 | 684 | | 13 (0.1-127)^b^;  164 (147-177)^e^ | 1105 (238-2412)^e^; | 4-20^h^ |  |
| **CXCL9**  **/MIG** | 575 | 792 | | 278 (139–540)^a^;  393 (228-676)^e^;  420(243-632)^k^ | 100-1600^d^;  2523 (1141-3821)^e^ | 1800-3000^h^ | 1930(710-4529)^k^ |
| **CXCL10**  **/IP-10** | 1046 | 35164 | | 576 (369–809)^a^;  804 (550–1073)^k^ | 100-1200^d^;  3486 (576-6348)^e^ | 2500-3500^h^ | 260-1700^j^  3555 (2077-17733)^k^ |
| **CXCL11**  **/I-TAC** | 23 | 760 | |  | 200-1600^d^ |  |  |

^a^(Kleiner et al. 2013); ^b^(Kim et al. 2011); ^c^(Maeda et al. 1992); ^d^(Singh et al. 2007); ^e^(Kleiner et al. 2015);

^f^(Andreasen et al. 2008), Escherichia coli 0113:H10:K at 0.3 ng/kg body weight, 2-3 hour post LPS injection;

^g^(van Deventer et al. 1990), Escherichia coli 0113:H10:K at 2ng/kg body weight, 1-3 hour post LPS injection;

^h^(Lauw et al. 2000), Escherichia coli standard lot G, at 4 ng/kg body weight, 5-6 hour post LPS injection;

^i^(Pillay et al. 2010), Escherichia coli standard lot Ec-5, at 2 ng/kg body weight, 1-2 hour post LPS injection;

^j^(Chan and Gu 2011); ^k^(Ng et al. 2007), Polymicrobial infection in preterm infants

**References:**

Anders S, Pyl PT, Huber W. 2015. HTSeq--a Python framework to work with high-throughput sequencing data. Bioinformatics 31(2):166-9.

Andreasen AS, Krabbe KS, Krogh-Madsen R, Taudorf S, Pedersen BK, Moller K. 2008. Human Endotoxemia as a Model of Systemic Inflammation. Current Medicinal Chemistry 15(17):1697-1705.

Brown RP, Delp MD, Lindstedt SL, Rhomberg LR, Beliles RP. 1997. Physiological parameter values for physiologically based pharmacokinetic models. Toxicol Ind Health 13(4):407-84.

Chan T, Gu F. 2011. Early diagnosis of sepsis using serum biomarkers. Expert Review of Molecular Diagnostics 11(5):487-496.

Hall RL, Miller RJ, Peatfield AC, Richardson PS, Williams I, Lampert I. 1980. A colorimetric assay for mucous glycoproteins using Alcian Blue. Biochemical Society Transactions 8(1):72-72.

Kim D, Pertea G, Trapnell C, Pimentel H, Kelley R, Salzberg SL. 2013. TopHat2: accurate alignment of transcriptomes in the presence of insertions, deletions and gene fusions. Genome Biol 14(4):R36.

Kim HO, Kim H-S, Youn J-C, Shin E-C, Park S. 2011. Serum cytokine profiles in healthy young and elderly population assessed using multiplexed bead-based immunoassays. Journal of Translational Medicine 9(1):113.

Kleiner G, Marcuzzi A, Zanin V, Monasta L, Zauli G. 2013. Cytokine levels in the serum of healthy subjects. Mediators Inflamm 2013:434010.

Kleiner G, Zanin V, Monasta L, Crovella S, Caruso L, Milani D, Marcuzzi A. 2015. Pediatric patients with inflammatory bowel disease exhibit increased serum levels of proinflammatory cytokines and chemokines, but decreased circulating levels of macrophage inhibitory protein-1beta, interleukin-2 and interleukin-17. Exp Ther Med 9(6):2047-2052.

Lauw FN, Pajkrt D, Hack CE, Kurimoto M, van Deventer SJ, van der Poll T. 2000. Proinflammatory effects of IL-10 during human endotoxemia. J Immunol 165(5):2783-9.

Love MI, Huber W, Anders S. 2014. Moderated estimation of fold change and dispersion for RNA-seq data with DESeq2. Genome Biol 15(12):550.

Maeda M, Watanabe N, Neda H, Yamauchi N, Okamoto T, Sasaki H, Tsuji Y, Akiyama S, Tsuji N, Niitsu Y. 1992. Serum tumor necrosis factor activity in inflammatory bowel disease. Immunopharmacol Immunotoxicol 14(3):451-61.

Merico D, Isserlin R, Stueker O, Emili A, Bader GD. 2010. Enrichment map: a network-based method for gene-set enrichment visualization and interpretation. PLoS One 5(11):e13984.

Ng PC, Li K, Chui KM, Leung TF, Wong RPO, Chu WCW, Wong E, Fok TF. 2007. IP-10 Is an Early Diagnostic Marker for Identification of Late-Onset Bacterial Infection in Preterm Infants. Pediatr Res 61(1):93-98.

Pillai VC, Strom SC, Caritis SN, Venkataramanan R. 2013. A sensitive and specific CYP cocktail assay for the simultaneous assessment of human cytochrome P450 activities in primary cultures of human hepatocytes using LC-MS/MS. J Pharm Biomed Anal 74:126-32.

Pillay J, Ramakers BP, Kamp VM, Loi AL, Lam SW, Hietbrink F, Leenen LP, Tool AT, Pickkers P, Koenderman L. 2010. Functional heterogeneity and differential priming of circulating neutrophils in human experimental endotoxemia. J Leukoc Biol 88(1):211-20.

Singh UP, Venkataraman C, Singh R, Lillard JW, Jr. 2007. CXCR3 axis: role in inflammatory bowel disease and its therapeutic implication. Endocr Metab Immune Disord Drug Targets 7(2):111-23.

Subramanian A, Tamayo P, Mootha VK, Mukherjee S, Ebert BL, Gillette MA, Paulovich A, Pomeroy SL, Golub TR, Lander ES and others. 2005. Gene set enrichment analysis: A knowledge-based approach for interpreting genome-wide expression profiles. Proceedings of the National Academy of Sciences 102(43):15545-15550.

van Deventer SJ, Buller HR, ten Cate JW, Aarden LA, Hack CE, Sturk A. 1990. Experimental endotoxemia in humans: analysis of cytokine release and coagulation, fibrinolytic, and complement pathways. Blood 76(12):2520-6.

Young MD, Wakefield MJ, Smyth GK, Oshlack A. 2010. Gene ontology analysis for RNA-seq: accounting for selection bias. Genome Biol 11(2):R14.

Yu J, Cilfone NA, Large EM, Sarkar U, Wishnok JS, Tannenbaum SR, Hughes DJ, Lauffenburger DA, Griffith LG, Stokes CL and others. 2015. Quantitative Systems Pharmacology Approaches Applied to Microphysiological Systems (MPS): Data Interpretation and Multi-MPS Integration. CPT Pharmacometrics Syst Pharmacol 4(10):585-94.
